# Early micro- and nanoscale microglial responses to blood–brain barrier modulation by transcranial focused ultrasound

**DOI:** 10.7150/thno.131051

**Published:** 2026-09-18

**Authors:** Elisa Gonçalves de Andrade, Jared VanderZwaag, Rikke Hahn Kofoed, Katherine Picard, Keelin Henderson Pekarik, Micaël Carrier, Fernando González Ibáñez, Mohammadparsa Khakpour, Kullervo Hynynen, Isabelle Aubert, Marie-Ève Tremblay

**Affiliations:** 1 Neuroscience Graduate Program, University of Victoria, Victoria, BC, Canada.; 2 School of Medical Sciences, Faculty of Health, University of Victoria, Victoria, BC, Canada.; 3 Department of Neurosurgery, Department of Clinical Medicine, Aarhus University, Aarhus, Denmark.; 4 Center for Experimental Neuroscience-CENSE, Department of Neurosurgery, Aarhus University Hospital, Aarhus, Denmark.; 5 Biological Sciences, Hurvitz Brain Sciences Research Program, Sunnybrook Research Institute, Toronto, ON, Canada.; 6 Axe neurosciences, Centre de recherche du CHU de Québec, Université Laval, Québec City, QC, Canada.; 7 Temerty Chair in Focused Ultrasound Research, Sunnybrook Health Sciences Centre and University of Toronto, Toronto, ON, Canada.; 8 Professor, Department of Medical Biophysics, University of Toronto, Toronto, ON, Canada.; 9 Cross Appointed Professor, Institute of Biomedical Engineering, University of Toronto, Toronto, ON, Canada.; 10 Department of Laboratory Medicine and Pathobiology, Temerty Faculty of Medicine, University of Toronto, Toronto, ON, Canada.; 11 Department of Biochemistry and Molecular Biology, University of British Columbia, Vancouver, BC, Canada.; 12 Centre for Advanced Materials and Related Technology (CAMTEC) and Institute on Aging and Lifelong Health (IALH), University of Victoria, Victoria, BC, Canada.

**Keywords:** microglia, blood-brain barrier modulation, focused ultrasound, scanning electron microscopy, ultrastructure

## Abstract

Modulation of the blood-brain barrier (BBB) using transcranial focused ultrasound with intravenous microbubbles (FUS) has progressed to clinical trials. Guided by magnetic resonance imaging (MRI), FUS significantly increases local BBB permeability for approximately 6-24 h in mice, with an ensuing mild inflammation. Microglia, as the immune cells of the brain, are stimulated by FUS-BBB modulation, although the timing and nature of their responses remain unclear. In this study, we characterized the cellular (i.e., density, distribution, and morphology) and subcellular (i.e., ultrastructure) microglial alterations early after FUS-BBB modulation.

**Methods:**

We targeted the hippocampi of adult, male mice with MRI-guided FUS in the presence of intravenous microbubbles. At 1 h and 24 h after FUS-BBB modulation, we investigated i) the density and distribution of ionized calcium-binding adaptor molecule 1 (Iba1)-positive (^+^) microglia; ii) the morphology of Iba1^+^ soma and processes; and iii) the microglial organelles and contacts using scanning electron microscopy.

**Results:**

Microglial density and distribution were unchanged at 1 h and 24 h. However, FUS-BBB modulation was associated with elongated microglial cell bodies at both time points. At 1 h, microglia shifted to compact shapes with stubby processes, whereas at 24 h, they were larger, with fewer processes. At the nanoscale, microglia preserved interactions with blood vessels, but they increased their contacts with swollen astrocytic endfeet at 1 h and 24 h. By 24 h after FUS-BBB, microglia contacted pre-synaptic elements and extracellular space pockets less frequently, with ultrastructural indications of changes in metabolic demand and lysosomal activity.

**Conclusion:**

At 1 h and 24 h after FUS-BBB, traits of microglial surveillance were maintained, despite a shift in morphology associated with injury shielding in a subset of cells. FUS-BBB modulation appears to temporarily modify lysosomal, but not phagocytic properties, of microglia and to reduce ultrastructural indications of pre-synaptic remodeling early after treatment.

## Introduction

Microglia are the resident immune cells of the central nervous system (CNS) 1,2. Changes in microglial activity, commonly known as reactivity, can alter CNS surveillance, synaptic remodeling, and blood-brain barrier (BBB) maintenance 3,4. Microglia coexist in a spectrum of states differing in their molecular expression, ultrastructure, morphology, distribution, and density 3-5. These levels of complexity determine microglial functions 4, making their detailed characterization necessary for identifying strategies to retain beneficial physiological roles.

Transcranial focused ultrasound combined with intravenously injected microbubbles (FUS) is a cutting-edge technology that modulates the BBB, and improves numerous neurological conditions 6-8. The BBB is a dynamic border checkpoint of the CNS, regulating the flux of molecules and cells 9-11. FUS transiently increases BBB permeability, improving the entry of therapeutic drugs into the CNS of rodents 12, non-human primates 13 and humans undergoing clinical trials 12.

In rodent models of neurodegenerative diseases, FUS-BBB modulation has been associated with beneficial microglial responses days after treatment, possibly facilitating the phagocytosis of deleterious molecules 14. In the healthy brain, reduced microglial proximity and cellular processes were found in the targeted compared to non-targeted hippocampi of mice, 7 days after FUS-BBB modulation 15. Evidence regarding microglial changes early after FUS-BBB modulation is lacking. Moreover, transient shifts in microglial morphology associated with reduced homeostatic parenchymal surveillance may be a contentious point of FUS-BBB modulation 8.

During acute inflammation, microglia shift their ultrastructure and present increased markers of cellular stress 16, including dilated endoplasmic reticulum (ER)/Golgi cisternae and enlarged mitochondria 17,18. Microglia showing ultrastructural markers of stress in neurodegenerative mouse models interact less with the vasculature 19, and healthy microglia seem to be required for timely closure of the BBB following FUS 20. Thus, during the window of FUS-induced BBB permeability, microglia under cellular stress could alter their physiological functions and impact the resolution of BBB properties involving other cellular players.

To provide insights into the early changes in microglial features, including surveillance and phagocytosis, following FUS-BBB modulation in adult male mice, we targeted the hippocampus, a memory and learning hub. We collected brains at 1 h and 24 h post-FUS-BBB modulation following a protocol previously shown to increase BBB permeability from 4 to 6 h in mice 10,21. Sections were stained for ionized calcium-binding adaptor molecule 1 (Iba1), a microglia/macrophage marker, and immunoglobulin (Ig)G, which we used as an indicator of increased BBB permeability 14,22. Using brightfield microscopy, we measured the density, distribution, and morphology of Iba1^+^ cells. With chip mapping scanning electron microscopy (SEM), we assessed microglial intracellular organelles (e.g., phagosomes) and contacts with the BBB and neuronal elements.

In male mice, our findings support that FUS-BBB modulation does not modify the density and distribution of hippocampal microglia in the first 24 h of treatment. Microglial cell bodies were enlarged, and a subset of cells shifted to larger shapes, with fewer processes. Moreover, there were more frequent microglial contacts with vessels presenting swollen astrocytic endfeet, but reduced microglial interactions with presynaptic elements and extracellular space pockets. In addition, as metabolic demand features increased (i.e., elevated mitochondrial alterations), microglia appeared to also reduce their lysosomal efficiency. Therefore, our results suggest that FUS-BBB modulation does not disrupt measures of microglial surveillance and phagocytosis and that a subset of cells adapts to BBB permeability, with potential effects on synaptic plasticity and other physiological roles.

## Results

### FUS-BBB modulation increased hippocampal BBB permeability

Given their roles in vascular regulation, BBB surveillance, and response to blood antigens, we examined the early responses of microglia to FUS-BBB modulation 8. Adult mice (n = 6 animals) received unilateral hippocampal FUS, with the contralateral region serving as a control. Post-FUS, gadolinium-enhanced MRI confirmed increased BBB permeability (Figure 1A, dashed circle), without significant differences in average voxel intensity ipsilaterally at 1 h and 24 h (Figure 1B, Table S1). We assessed permeability *post-mortem* with IgG immunostaining 14,22, observing it exclusively in the ipsilateral hippocampi (Figure 1C), particularly in the *cornu ammonis 1 (CA1)*. Accordingly, IgG staining optical density increased in the 24 h ipsilateral hemisphere compared to the 1 h ipsilateral hemisphere, and the 1 h and 24 h contralateral hemispheres post-FUS-BBB modulation (Figure 1D, main effect, F = 17.49 and *p =* 0.0001, post-hoc contralateral 1 h *vs* ipsilateral 24 h *p ≤* 0.0001, post-hoc ipsilateral 1 h *vs* ipsilateral 24 h *p ≤* 0.0001, post-hoc contralateral 24 h *vs* ipsilateral 24 h *p ≤* 0.0001, Table S1). The *CA1* is organized in four strata with distinct neuronal, microglial and vasculature compositions, but there was no significant difference in IgG optical density across layers (Table S1). These results suggest that FUS-BBB increases hippocampal permeability, enabling gadolinium and IgG entry in the male mouse brain parenchyma.

### Microglial density and distribution were maintained

Microglia may rearrange their distribution over 1 and 3 days 20 or 7 weeks 15,23 after FUS-BBB modulation. To assess earlier effects, we quantified Iba1^+^ cell density, cluster cell density (<12 µm apart), nearest neighbour distance (NND, nearest cell to every other cell), and spacing index (NDD and density compiled) in ipsilateral and contralateral *CA1* layers at 1 h and 24 h (Figure 2A-D). No significant differences were found between hemispheres in any layer (Figure 2E-F, Table S2). In the pooled *CA1* layers, there was a significant effect of time in modulating the NND (Figure 2G, main effect, F = 5.5390, p = 0.0464), indicating the distance between cells changed over the 24 h after FUS-BBB modulation.

### Microglial density was inversely correlated with parenchymal IgG

Because microglial somas and processes may be attracted to blood molecules extravasation 8, we correlated parenchymal IgG optical density and microglial properties in the ipsilateral *CA1*. At 1 h and 24 h, IgG optical density correlated negatively with microglial density (Figure S1A, r = -0.94 and *p* = 0.02, Table S3) and positively with microglial NND (Figure S1A, r = 0.89 and *p* = 0.03, Table S3). These results suggest that the quantity of IgG entry into the brain was associated with decreases in density and proximity of Iba1^+^ cells in male mice. Longitudinal imaging of microglia during the hours following FUS-BBB modulation is warranted to verify this association 24,25.

### Microglial soma perimeter increased

Microglia soma enlargement occurs 7 days after FUS-BBB modulation in male mice 15. We tested whether earlier changes appear in the *CA1 stratum lacunosum-moleculare* (*LMol*), a vessel-rich layer 26 possibly more affected by FUS 27. Manual tracing of cell bodies (Figure 3A-D) revealed an increased average soma perimeter ipsilaterally at 24 h (Figure 3E, main effect F = 15.4300 and *p =* 0.0171, interaction effect F = 8.0070 and *p =* 0.0474, post-hoc contralateral *vs* ipsilateral at 24 h *p =* 0.0175 and *t* = 4.7780, Table S4), without changes in soma area, aspect ratio, solidity, roundness, or circularity (Figure 3F-J, Table S4). Thus, the microglial soma perimeter increased by 24 h of FUS-BBB modulation, producing elongated or complex cell body shapes.

To capture subtle morphological shifts across the cell population 4, we analyzed the distribution of soma descriptors via nonlinear regression (See Supplementary Methods for a detailed description) 28,29. The ipsilateral distribution of microglial soma perimeter, area, aspect ratio, solidity, and roundness was significantly different from controls at both time points (Figure 3K-O, all *p* < 0.0001), though only at 1 h for soma circularity (Figure 3P, *p* < 0.0001). Based on the amplitude and mean of the regression curves, which increase with less heterogeneous but larger values across cells, ipsilateral microglia deviated to larger, rounder and more porous somas at 1 h and 24 h, resulting in diverse distributions for all shape descriptors at 24 h (Table S5). Our findings indicate FUS-BBB modulation in male mice increased soma perimeter broadly and promoted large and varied microglial somas after 1 h and 24 h.

### Microglial processes shifted in a subset of cells

In addition to soma changes, FUS-BBB modulation may influence microglial processes, which remodel to survey cellular compartments and extracellular space 3*.* After tracing *LMol* Iba1^+^ staining to define convex territories and process masks (Figure 4A-D), we measured convex and mask area, perimeter, aspect ratio, circularity, and solidity, along with descriptors that increase with cell shape complexity, i.e. lacunarity and fractal dimension (Table S4) 30. None of these averaged descriptors differed between hemispheres and time points (Figure 4E-J). However, nonlinear regression revealed significant ipsilateral shifts in the relative distribution of all microglial process shape descriptors at 1 h and 24 h, except for microglial area at 1 h and circularity at 24 h (Figure 4K-P, Table S5, all *p <* 0.02). Ipsilateral microglia adopted a diverse distribution of smaller and regular process shapes at 1 h, shifting back to a relatively homogeneous distribution of larger, compact and irregular shapes at 24 h (Supplementary Methods). Thus, in a subset of cells, FUS-BBB modulation promoted a diverse set of de-ramified (from ramified to rounder) process morphologies at 1 h and of porous morphologies at 24 h (Table S5).

When we examined microglial ramification further by skeletonizing Iba1^+^ masks (Figure 5A-D), none of the average descriptors associated with increased process ramification, i.e., number of branches and junctions, maximum branch length and the longest shortest path, changed between hemispheres and time points (Figure 5E-H, Table S4). However, there were significant shifts in their ipsilateral relative distribution at 1 h and 24 h (Figure 5I-L, Table S5, all *p* < 0.0451). Ipsilateral microglial cells had a relatively homogeneous distribution of numerous but smaller branches at 1 h, shifting back to less ramified and elongated processes at 24 h (Supplementary Methods). In summary, FUS-BBB modulation did not cause large-scale changes in microglial territory or process shape at 1 h or 24 h (Table S5). Instead, subsets of ipsilateral cells shifted their morphology, adopting diverse distributions of larger and elongated somas, as well as compact territories with stubby processes at 1 h. At 24 h, this reverted to homogeneous distributions of larger territories with fewer and simpler processes (Table 1).

### Microglial morphology was not linked to parenchymal IgG

We tested whether ipsilateral microglial morphological variation correlated with IgG extravasation into the parenchyma at both time points 8. Spearman r correlations between the IgG staining optical density and microglial shape descriptors showed no significant relationships (Figure S1B, Table S3), suggesting that the levels of IgG were not directly linked with shifts in microglial shape in male mice.

To assess variability and the contribution of shape descriptors to microglial morphology, we subjected all 30 ipsilateral and contralateral shape variables to a principal component analysis (PCA, Table S7). Six PCs were retained, with PC1 explaining 39.2 and PC2 13.9% of the variation in the dataset. Ipsilateral microglia at 24 h *vs* 1 h accounted for much of this variability (Figure S1C), and arborization parameters such as number of branches and junctions most strongly correlated with PC1 variation (Figure S1D).

### Microglia selectively contacted swollen astrocytic endfeet

Electron microscopy has shown that microglia interact with 31,32 and can migrate toward vessels upon BBB permeability 4,33,34. This raised the intriguing possibility that after FUS-BBB, microglia increase their vascular contacts, particularly in large-vessel regions such as the *LMol* 26. Using chip mapping SEM, we quantified the interactions between Iba1^+^ cells and blood vessels, including capillaries, veins and arteries (6A-D). While we observed one striking case of an ipsilateral Iba1^+^ process enclosed by a capillary at 1 h (Figure 6B), average vessel distance (Figure 6E) and number of capillary-associated microglia (within <150 nm of a vessel) did not differ at 1 h and 24 h (Table S8). Instead, FUS-BBB modulation may promote microglial probing of specific BBB elements 8. First, we verified microglial cell body interactions with the basement membrane (Figure 6A-D), an extracellular matrix that anchors BBB cells 8. We found a significant interaction between hemisphere and time for microglial-basement membrane contacts (Figure 6F, interaction effect, F = 12.31, *p* = 0.02). The absence of significant pairwise differences suggests the overall effect is driven by many small, coordinated changes in these contacts, rather than one dominant pairwise difference.

Next, we examined microglial cell body contacts with astrocytic endfeet, which link neuronal activity to blood flow 8. Despite many interactions (Figure 6A-D), contact frequencies were unchanged across conditions (Figure 6G). Notably, swollen endfeet, which occur in health and disease and are marked by large and clear cytoplasm 35, were observed in both hemispheres (Figure 6B). There was a significant hemisphere effect on the number of microglia-swollen endfeet contacts (Figure 6H, main effect, F =16.48, *p* = 0.02) and an increased frequency of these interactions ipsilaterally at both time points (Figure 6I, 1 h *p =* 0.0007, 24 h *p =* 0.0101, Table S9). Our findings propose a selective recruitment of microglia towards swollen endfeet after FUS-BBB modulation in male mice.

### Microglia preferentially interacted with larger and nearby vessels

We also tested whether vessel proximity and diameter correlated with microglial-BBB interactions (Figure S2A, Table S12). We found negative correlations between vessel proximity and microglial contacts with the basement membrane (r = -0.4722, *p ≤* 0.0001), homeostatic astrocytic endfeet (r = -0.7330, *p ≤* 0.0001), swollen astrocytic endfeet (r = -0.3126, *p* = 0.0019), and total astrocytic endfeet containing mitochondria (Figure 6C-D, r = -0.6213, *p* = 0.0000), which were shown to attract immune cells 36. By contrast, there were positive correlations between vessel area and microglial interactions with homeostatic astrocytic endfeet (r = 0.3624, *p ≤* 0.0001), swollen endfeet (r = 0.3072, *p* = 0.0001), astrocytic endfeet containing mitochondria (r = 0.3151, *p* = 0.0001), the basement membrane (r = 0.1793, *p* = 0.0276), as well as endothelial cells (Figure 6A-B, r = 0.1800, *p* = 0.0270). Therefore, microglial cell bodies in male mice appear to preferentially interact with larger, nearby vessels, particularly at swollen endfeet, after FUS-BBB modulation.

### A subset of microglia reduced contacts with pre-synaptic elements and extracellular space pockets

The role of microglia in synaptic plasticity is further supported by previous electron microscopy studies in animal models 37. Since FUS-BBB modulation may trigger synaptic plasticity 8, we used chip mapping SEM to examine interactions between microglial cell bodies, pre-synaptic (Figure 7A-B) and post-synaptic (Figure 7A-D) elements, and extracellular space pockets (Figure 7A-C), required for neuronal remodeling 38,39. Time modulated the average number of microglia-pre-synaptic contacts (Figure 7E, main effect, F = 33.0600, *p =* 0.00), suggesting their number changed over 24 h. Across the cell population, the relative distribution of microglia-pre-synaptic contacts shifted between hemispheres at 1 h and 24 h (Figure 7F all *p ≤* 0.0001), with ipsilateral microglia showing fewer and variably distributed contacts (Supplementary Methods, Table S10). Ipsilateral microglia also contacted extracellular space pockets less frequently at 24 h after FUS-BBB modulation (Figure 7H, *p =*0.0040, Table S9). We did not find significant differences for microglial interactions with post-synaptic elements, neuronal cell bodies (satellite cells), myelinated axons, degenerating myelin, and extracellular space pockets containing debris or presenting digestion (Table S8). These results suggest a subset of the mouse microglial population shifts towards less frequent contacts with pre-synaptic elements and extracellular space pockets, potentially reflecting a reduced contribution of microglia to synaptic plasticity after FUS-BBB modulation 8.

### Markers of microglial oxidative stress increased in a subset of cells

Microglial organelle density and ultrastructure can indicate oxidative stress after BBB permeability 35. Using chip mapping SEM, we examined ER/Golgi and mitochondria density (Figure 8A-D), as well as hallmarks of metabolic demand, such as ER/Golgi dilation, mitochondria elongation (Figure 8B) and dystrophy (Figure 8D) in microglial cell bodies 30. There were no significant differences in microglial densities of ER/Golgi cisternae and mitochondria at 1 h or 24 h (Figure 8E-H), despite hemisphere-dependent effects on the number of homeostatic (Figure 8F, main effect, F = 17.3200, *p =* 0.0141) and dystrophic (Figure 8H, main effect, F = 5.5910, *p =* 0.0456) mitochondria (Table S8). The relative distributions of ER/Golgi cisternae and of homeostatic and elongated mitochondria significantly differed between hemispheres at 1 h and 24 h (Figure 8I-K, all *p ≤* 0.0001, Table S10). At 1 h, ipsilateral microglia exhibited organelle changes indicative of higher metabolic demands, with an increasingly heterogeneous ER/Golgi distribution, but the reverse effect for homeostatic and elongated mitochondria (Supplementary Methods). At 24 h, a higher frequency of ipsilateral microglia contained dystrophic mitochondria (Figure 8L, *p =* 0.0040, Table S9). In summary, FUS-BBB modulation was associated with oxidative stress markers in a subset of microglia, revealed by increased ER/Golgi activity and mitochondrial alterations.

### Alterations to lysosomal properties in microglia

FUS-BBB modulation has been linked to microglial phagolysosomal activity, based on an increased inclusion of pathological proteins 14,40. We used chip mapping SEM to quantify the number of empty and filled phagosomes, autophagosomes and primary (Figure 9A), secondary (Figure 9B), and tertiary (Figure 9C) lysosomes within microglial cell bodies (Figure 9D) following FUS-BBB modulation. We found significantly fewer primary lysosomes per microglia at 24 h (Figure 9E, interaction effect of hemisphere x time F = 12.7200 and *p =* 0.0235, post-hoc contralateral *vs* ipsilateral at 24 h *p =* 0.0368 and t = 3.8340). Secondary lysosomes also showed a significant interaction between hemisphere and time (Figure 9F, interaction effect, F = 8.6430 and *p =* 0.0424). Since no individual pairwise contrast reached significance, this result indicates a group-level pattern rather than a singular large difference. No significant differences were found for tertiary lysosomes (Figure 9G). Accordingly, there were fewer microglial cell bodies with primary (Figure 9H, *p =* 0.0259) and secondary lysosomes at 24 h (Figure 9I, *p =* 0.0379), but no impact on tertiary lysosomes (Figure 9J, Table S9). Primary and secondary microglial lysosomal activity decreased 24 h after FUS-BBB independently of phagocytosis, as we did not find significant differences for the average number and relative frequency of empty and filled phagosomes, and autophagosomes (Tables S8-9).

### Microglial ultrastructure variability was higher at 24 h

Modifications in microglial contacts with blood vessels and the neuropil can coincide with changes in their organelles. To address the multidimensionality and contribution of each feature to ipsilateral microglial ultrastructure, we explored all 30 ultrastructural variables using PCA (Table S11). The analysis selected three PC, with PC1 explaining 13.7% and PC2 11.05% of the variance in the dataset. Ipsilateral microglia at 24 h accounted for most variability (Figure S2C). Parameters such as the number of contacts between microglial cell bodies and astrocytic endfeet, swollen astrocytic endfeet, basement membrane, along with alterations in ER/Golgi cisternae and mitochondria, show the strongest correlations with variation within PC1 and PC2 (Figure S2D and Table S11).

## Discussion

Several animal model reports have looked at microglial transcriptomic and morphological responses starting a day after FUS-BBB modulation 24,41-43, but similar evidence from the earlier hours of FUS-induced BBB permeability is still limited 8,14,15,23,43. Our study explored whether microglial properties change early on following FUS-BBB. We leveraged hippocampal FUS-BBB to investigate microglial density, distribution, morphology, and ultrastructure at peak BBB permeability and after its resolution at 1 h and 24 h, respectively. We found that microglia generally do not change their density and distribution in the early stages after FUS-BBB. Cellular morphology was largely stable, with subsets of cells shifting towards shapes that were compact and stubby at 1 h, as opposed to extensive and simple at 24 h. Microglia likely increased surveillance of blood vessels presenting astrocytic swelling at both time points, while microglial contacts with pre-synaptic elements and extracellular space pockets tended to decrease at 24 h. In parallel, microglial cell bodies exhibited increased dystrophic mitochondria and reduced primary lysosomes at 24 h, suggesting increased metabolic demand and alterations to lysosomal properties, such as trafficking, turnover, or digestive demands 44. Thus, we argue that by 24 h post FUS-induced BBB permeability there are no global changes in proxies of microglial surveillance and phagocytosis, raising important questions about their response over more chronic time periods.

To our knowledge, this is the first study to characterize early changes in microglial morphology quantitatively following FUS-BBB modulation. Only microglial soma perimeter was significantly increased in the treated hemisphere at 24 h after FUS-BBB. This finding contrasted with the elevated microglial soma area previously observed in cells adjacent to the targeted hippocampi a week after FUS-BBB 18. An early increase in perimeter not accompanied by changes in area could indicate an elongated soma shape reminiscent of rod microglia 45, which are proposed to shield dendrites and axons, notably after traumatic brain injury in rats 46. It could also be an indication of membrane ruffling associated with cellular motility, receptor internalization and other activities 47. Indeed, there was an increase in the relative distribution of large, round and porous microglial somas at 1 h and 24 h, based on nonlinear regression. Combined with the increased heterogeneity in the relative distribution of most microglial soma shape descriptors within the population at 24 h, we speculate that individual microglia could diversify their cell body shape, some ultimately adopting an elongated or complex morphology 24 h after FUS-BBB modulation.

Microglia can also use their processes to shield damage; only 30 min after a BBB lesion, cortical vessel-associated microglia were seen extending their processes to reduce BBB permeability in mice 48. Our findings suggest that FUS-BBB does not drive immediate and global changes in microglial ramification. However, microglia did shift their stubby processes at 1 h to larger, fewer and simpler processes at 24 h. Selective modulation of arborization aligns with early observations that microglia constantly modify their morphology, and this can be intensified with BBB damage 49. The shift towards de-ramification that we observed could support the rod-like state mentioned before; however, it is also likely that these cells adopt more than one type of arbour morphology in response to FUS-BBB 3. Cells transitioning from a ramified to an ameboid microglial morphology after lesion generally undergo withdrawal of processes, followed by soma enlargement and process protrusion 50. In the putamen of non-human primates, a qualitative increase in microglia was observed, associated with a decreased cell territory at 3 h and 7 days but not 30 days after FUS-BBB modulation 51. Hence, the heterogeneous relative distribution of process shape descriptors we observed within the microglial population at 1 h may also reflect a push to maintain the diverse, specialized roles of microglia, in parallel to a partial transition toward ameboid cells, which may serve a protective role in the parenchyma 8,50.

Following previous evidence of microglia dynamically approaching blood vessels 4,33,34, and potentially sealing BBB leakages 34,36, we did not identify significant differences in the average distance or number of microglial cell bodies associated with blood vessels at 1 h and 24 h after FUS-BBB. Similarly, our light microscopy findings suggested the distribution of microglia did not change, despite a main effect of time in modulating the overall distance between cells, or NND. A significantly reduced NND of hippocampal microglia was previously observed in mice one week after FUS-BBB modulation 15. Moreover, single-cell sequencing analysis of mouse hippocampal microglia at 24 h after FUS-BBB modulation revealed a molecular profile indicative of cell proliferation 20. It is plausible that changes in microglial density and distribution after FUS-BBB take longer than 24 h. Contrary to rapid process movements 48, rodent microglial cell bodies are much less mobile, both under healthy conditions and after BBB lesion 49, with only 7.5% of microglial cell bodies shifting >5 μm toward vessels over several days 52. We found that microglial cell bodies do not migrate toward vessels in the 24 h after FUS-BBB in mice, in contrast to the results obtained in non-human primates at a 48 h time point where migration was observed using light microscopy 42. Future studies considering FUS parameters, species, sex and time post-FUS are, therefore, warranted prior to generalization of the findings.

In addition, changes in microglial distribution may occur in relation to plasma extravasation into the CNS 53. In rodent studies where microglial cell bodies quickly approached vessels, e.g. after daily injection of lipopolysaccharide 34 or stroke 36, the BBB suffered more intense insults compared to a single FUS-BBB 6. Similar to previous studies, IgG from the blood, endogenous or administered intravenously, can be detected at the FUS-BBB treated sites following their entry and prior to their clearance from the parenchyma, which can take up to a week 14,54-58. Correspondingly, there was a correlation between IgG optical density and NND 1 h and 24 h after FUS-BBB, suggesting elevated BBB permeability pushes microglia closer to one another or to damaged areas. Plasma extravasation, in turn, may differ based on vessel type, as arterioles were proposed to undergo more active vesicular transport than capillaries and venules during FUS-BBB 9. Indeed, of all *CA1* strata, the *LMol*, with its abundant large vessels 59, was the main region to visually show parenchymal IgG at 1 h and 24 h. We also found a positive correlation between vessel area and interactions between microglia and BBB elements. Moreover, we observed a significantly higher frequency of microglial contacts with swollen astrocytic endfeet in the ipsilateral *vs* contralateral *LMol* at both 1 h and 24 h. Previously, FUS targeted at brain tumours was shown to increase astrocytic endfeet swelling in rats 60. However, swollen endfeet can occur naturally in the *LMol,* that is, in healthy, non-treated mice 43. Our findings suggest that larger vessels bearing swollen endfeet are likely to recruit microglia after FUS-BBB, necessitating further longitudinal analyses.

Increased recruitment of microglia towards vessels could hinder physiological surveillance of synapses and help explain the reduction in contacts between microglial cell bodies and pre-synaptic elements we observed for subsets of cells in the targeted *LMol*. Shifts in microglial morphology towards rod or ameboid cells are typically associated with reduced surveillance 31. Based on the current literature, reduced microglia pre-synaptic interactions may decrease instances of trogocytosis, a process by which microglia partially engulf pre-synaptic elements and extracellular matrix components 37,61. However, this interpretation remains speculative, as we did not empirically evaluate this mechanism. Accordingly, we observed decreased the frequency of microglial contacts with extracellular space pockets in the ipsilateral compared to contralateral *LMol* at 24 h after FUS-BBB, raising the possibility that FUS-BBB modulation has immediate effects on micro-surveillant activities in the parenchyma.

Following stereotaxic injection of plasma into the brain, microglia increased their expression of genes related to oxidative stress 53. Although restricted, due to a lack of vessel damage as in stereotaxic injections 8, penetration of plasma molecules into the CNS following FUS-BBB modulation may also induce cellular stress and impact the metabolism of microglia. We found that microglial mitochondrial damage was more frequent in the ipsilateral *vs* contralateral *LMol* 24 h after FUS-BBB, that is, while the number of microglial homeostatic mitochondria decreased with time in the ipsilateral hippocampi, the number of dystrophic microglia increased, as did their frequency per cell. This was supported by nonlinear regression models, where the distribution of microglial cells with ultrastructural markers of higher metabolic demands increased in the ipsilateral *LMol* at both time points. Accordingly, elevated [18F] DPA-714 binding to the translocator protein, non-selectively present in microglial mitochondria and typically upregulated with inflammation, was identified at 24 h after FUS-BBB in rat hippocampi 41. In our study, indications of mitochondrial stress were accompanied by significantly fewer immature lysosomes, but stable phagosome density at 24 h in the ipsilateral *LMol*. These findings suggest that microglia maintain regular phagocytic intake but may undergo acute alterations to cellular metabolism and organellar stress early after FUS-BBB. Sequencing of mouse hippocampal microglial 1 and 3 days after FUS-BBB indicated an enrichment for lysosomal and phagocytic pathways 20. Despite not observing changes in microglial phagosomal content, acute alterations to lysosomal properties early after FUS-BBB modulation indicate an acute metabolic response, which may stimulate alterations to phagocytic activity at later time-points. This microglial response is essential to maintaining homeostasis, and disruptions can contribute to brain pathology as observed through colocalization of microglial markers and misfolded proteins in Alzheimer’s disease pathology mouse models 8,14,62.

Overall, we show that within the microglial population, subsets of cells shift to elongated, less ramified shapes that potentially shield the brain parenchyma from blood-antigens or mechanical stress one day after FUS-BBB modulation. This shift is in line with more frequent interactions between microglia and vessels showing astrocytic alterations, as well as increased ultrastructural indications of microglial metabolic alterations and organellar stress. In parallel, microglia reduced their frequency of interactions with pre-synaptic elements and extracellular space pockets. Despite limitations to our work (discussed below), our findings suggest that some microglia are engaged in restoring the properties of the BBB instead of their roles in pre-synaptic plasticity, and astrocytes at the BBB are characterized by swollen astrocytic endfeet.

### Limitations

Our cellular and subcellular analyses provide a detailed characterization of early microglia changes after FUS-BBB modulation in male mice; however, long-term morphological and functional effects, remain to be evaluated. While we used a priori power calculations in every analysis, we acknowledge that larger cohorts are recommended in future studies. Increasing evidence demonstrates that microglia exhibit specific phenotypic responses in females compared to males, including elevated oxidative stress gene expression following BBB permeability in female mice 63. However, similar to the present study, previous research in the field used mainly male rodents 8. Future studies investigating female cohorts and sex differences are highly encouraged, as they may prove beneficial in improving therapeutic FUS-BBB modulation for the female population. Furthermore, it is clear that the effects of FUS + microbubbles, mechanical or otherwise, can contribute to, and extend beyond, changes in microglia and impact other cells of the neurovascular unit. For example, astrocytes can instruct microglia to seal astrocytic endfeet gaps within the BBB in certain contexts, such as during development 34,64. Also, several studies have provided evidence of astrocytic modulation following FUS-BBB [review [65]]. Our findings suggest astrocytic endfeet swelling may increase microglial contacts with the BBB. Thus, assessing the dynamic crosstalk between microglia and astrocytes, as well as the remaining cells of the neurovascular unit, is warranted to provide full mechanistic insight into FUS-BBB modulation 65. Additionally, BBB modulation was induced using relatively mild ultrasound exposures, and higher exposure levels would be expected to produce more pronounced microglial effects. Finally, throughout the experiments, Iba1 was used as a universal marker of resident microglia. However, Iba1 is also expressed by border-associated, perivascular, and circulating myeloid-lineage cells 66. Although the FUS-BBB modulation settings we utilized are not typically associated with macrophage infiltration, we cannot rule out the possible inclusion of non-microglia, myeloid-lineage cells in our analyses.

While this work is an important first step to understanding acute cellular response following FUS-BBB modulation, it remains to be seen if these findings differ across health and disease, throughout multiple brain regions and repeated treatments, all of which would be relevant to human conditions. In healthy or Alzheimer’s disease pathology mouse models, cortical, thalamic and hippocampal FUS-BBB modulation consistently resulted in increased Iba1 expression, despite inherent differences in the microenvironment and microglial populations, such as higher density and morphological ramification in the hippocampus and frontal brain, compared to the midbrain 8,67. Future work should explore the cumulative effects of FUS treatments across brain regions with the greatest translational impact, such as the hippocampus and cortex, and within both homeostatic and disease-related contexts to verify if there are more nuanced responses of microglial cells.

## Materials and Methods

### Animals and ethics

Male (n = 6 animals) C57BL/6J mice, 3 months of age, were purchased from the Jackson Laboratory and group-housed at 18-22 °C, 40-60% humidity and a 12 h light/dark cycle with* ad libitum* access to food and water. All the animal experiments were conducted following ethical approval from the Sunnybrook Research Institute Animal Care Committee and according to the Canadian Council on Animal Care Policies & Guidelines and the Animals for Research Act of Ontario (AUP788).

### Magnetic resonance imaging-guided focused ultrasound sonication with microbubbles

Mice were anaesthetized using isoflurane at 2–3% with medical air as the gas carrier for 30 min (CP0406V2, Fresenius Kabi, Toronto, Canada). Each mouse was placed in a supine position on an MRI-compatible sled, with an angiocatheter inserted into the tail vein. The sled was inserted in a 7.0 T MRI (BioSpin 7030, Bruker, Massachusetts, United States of America [USA]) used to target the ventral hippocampi in the left hemisphere (ipsilateral) with a single focus beam, while the right hemisphere (contralateral) served as a control. The ultrasound waves were generated by a 0.58 MHz spherically focused transducer (75 mm outer diameter, 26 mm inner diameter, 60 mm radius of curvature) driven at the 3rd harmonic (1.78 MHz) and applied for 120 s in 0.01 s bursts at a frequency of 1 Hz, while 0.02 mL/kg Definity® MB (Lantheus Medical Imaging, Massachusetts, USA) were injected via the angiocatheter. The acoustic pressure was increased incrementally after each burst until subharmonic emissions were detected, when the acoustic pressure was reduced to 50% and maintained throughout the ultrasound treatment 21. Subharmonic emissions were detected by a 16 mm diameter PZT hydrophone in the centre of the transducer and analyzed as described previously 68. Immediately after, animals were injected with the gadolinium-based MRI contrast agent Gadovist (Bayer Inc., Mississauga, ON, Canada) 69-71, followed by a 100 mL flush of 0.9% saline solution injected through the angiocatheter to ensure complete administration. Gadolinium-enhanced T1-weighted MRI scans were acquired with no delay to assess BBB permeability in real-time. The latter was quantified post-mortem using Medical Image and Processing, Analysis and Visualization software (V11.0.17 for Mac OS X 10.7, MIPAV, National Institutes of Health, Maryland, USA) and region-based analysis, as previously described 70. Briefly, a 1 × 1 mm^2^ square, the theoretical size of the focused ultrasound spot, was used to measure the average voxel intensity in each ipsilateral targeted and corresponding non-targeted contralateral brain region for all animals 70.

### Perfusion and tissue sectioning

After 1 h and 24 h post FUS, male (n = 3 animals/time point) adult mice were anaesthetized with a mix of ketamine (80 mg/kg)/xylazine (10 mg/kg) and transcardially perfused with phosphate-buffered saline (PBS; 50 mM, pH 7.4), followed by 3.5% acrolein and 4% paraformaldehyde diluted in phosphate buffer (PB; 100 mM, pH 7.4). Brains were post-fixed in 4% paraformaldehyde diluted in PBS for 2 h at 4°C, and the ipsilateral and contralateral brain hemispheres were separated. 50 μm longitudinal brain sections were next prepared in ice-cold PBS using a vibratome (VT1200S, Leica Biosystems, Ontario, Canada) at a frequency of 90–100 Hz and a speed of 0.5 mm/s. The sections were stored at -20°C in cryoprotectant (30% (v/v) glycerol and 30% (v/v) ethylene glycol in PBS) until further processing.

### Double immunohistochemistry and brightfield imaging

Double immunohistochemistry staining against Iba1 and IgG was performed in 3 sections containing the ventral hippocampi (Bregma -2.36 mm to -3.44 mm) from the ipsilateral and contralateral hemispheres per animal (n = 3 animals/time point) 72. Sections were first assessed for IgG staining, used to delineate regions of increased BBB permeability after FUS-BBB modulation 14. Briefly, free-floating sections were washed in PBS and quenched with 2% H_2_O_2_ diluted in 70% methanol for 10 min, followed by 0.1% NaBH_4_ diluted in PBS for 30 min. After 3 additional PBS washes, sections were incubated in a blocking solution of 10% normal donkey serum and 1% Triton X-100 in Tris-buffered saline (TBS; 50 mM, pH 7.4) for 1 h at room temperature (RT) and subsequently with donkey anti-mouse IgG secondary antibody (1:500 in blocking buffer, cat# 715-065-150, Jackson ImmunoResearch, Philadelphia, USA) for 2 h at RT. Following 3 washes in TBS, the sections were immersed in an avidin-biotin solution (1:100 in TBS, cat# VECTPK6100, VECTASTAIN, Vector Labs, California, USA) for 1 h at RT. The staining was next revealed by incubation with a HRP substrate kit solution (cat# SK-4600, Vector® VIP, Vector Labs, California, USA). The next day, the sections were stained for the marker Iba1, which is expressed by microglia in the brain parenchyma, as well as peripheral macrophages and border-associated macrophages 3. It should be noted that the FUS-BBB modulation settings we utilized are not typically associated with peripheral immune cell infiltration into the brain 8, and only parenchymal cells were analyzed. We thus designated Iba1^+^ cells as microglia across the manuscript. Briefly, for Iba1 staining, sections were immersed in a blocking solution of 10% fetal bovine serum, 3% bovine serum albumin and 1% Triton X-100 in TBS for 1 h at RT. Subsequently, the sections were incubated with a rabbit anti-Iba1 primary antibody (1:1000 in blocking buffer, cat# 019-19741, FUJIFILM Wako Chemical, Virginia, USA) overnight at 4°C. The following day, after 3 consecutive TBS washes, the sections were incubated in a biotinylated goat anti-rabbit secondary antibody (1:300 in TBS, cat# 111-066-046, Jackson Immunoresearch) and subsequently in an avidin–biotin solution for 1 h. Staining was revealed with 0.05% 3-3′-diaminobenzidine (DAB, D5905-50TAB, Millipore Sigma, Massachusetts, USA) and 0.015% H_2_O_2_ in 100 mM Tris–HCl. After mounting and drying, the sections were incubated in ultrapure H_2_O, increasing ethanol concentrations (50%, 70%, 80%, 90%, 100%) and xylene (cat# 534056, Millipore Sigma) for 5 min each at RT. The glass slides were cover-slipped with distyrene, plasticizer, and xylene mounting medium (cat# 13510, Electron Microscopy Sciences, Pennsylvania, USA). Each hemisphere was imaged (n = 3 animals/time point) in a single z plane at 40× with a numerical aperture of 0.65, using a Basler area scan camera (2.3 MP; acA1920-40uc) and visualized through the Microvisioneer manualWSI Scan software (2019B-3S; Microvisioneer, Baden-Württemberg, Germany).

### IgG qualitative and quantitative brightfield analysis

All analyses were performed blinded to the experimental conditions using FIJI software (V2.13.1 for Mac OS X 10.7, FIJI, National Institutes of Health, Wisconsin, USA) 73, as previously described 38. First, the freehand selection tool was used to trace the regions of interest (ROI), i.e. *CA1* and its strata, *Or*,* Py*, *Rad*,* LMol* 72. Considering the structural diversity within these layers 74,75, each stratum was analyzed individually. Next, the IgG staining was visually identified in the ROI through Vector® VIP substrate deposition and measured in 3 sections (in both the ipsilateral and contralateral hemispheres per animal; n = 3 animals/time point), using an optical density analysis, as previously described 49.

### Microglial density and distribution analyses

The Iba1^+^ cell density and distribution in the *CA1* and its strata were assessed in 3 sections (in both the ipsilateral and contralateral hemispheres per animal; n = 3 animals/time point) via a semi-automated macro. In total, 3,079 Iba1^+^ cells (minimum of 227 microglia/layer/time point), were counted, a sample size which was sufficient to obtain statistical power based on calculations obtained using G*Power software (V3.1.9.6 for Mac OS X 10.7, G* Power Software, Nordrhein-Westfalen, Germany) (effect size of 0.25 and power of 0.8 estimated to 128 individual cells) 76. These numbers are consistent with previous literature in the field 39,77,78. Briefly, each Iba1^+^ cell in focus presenting a minimum of 3 processes was marked with the brush tool. Next, the ROIs were processed utilizing the grayscale, the thresholding and the plugins “analyze particles” as well as “nearest neighbour distance, NND”. Microglial density (# microglia/μm^2^) was defined as the total number of Iba1^+^ cells divided by the area. The NND (μm) was obtained by quantifying the average distance between each Iba1^+^ cell to its nearest neighbour. The spacing index (arbitrary unit, a.u.) was calculated as the square of the average NND multiplied by the microglia density. Two or more cells less than 12 µm apart were considered a cluster and averaged by animal to obtain the cluster density (# clusters/μm^2^) 75.

### Microglial morphology analysis

*LMol* Iba1^+^ cells were randomly selected in 3 sections (in both the ipsilateral and contralateral hemispheres) (n = 21 cells/hemisphere, N = 3 animals/time point) for the morphology analysis. This sample size was sufficient to obtain statistical power based on calculations using the G*Power software 76 and is consistent with previous literature in the field 39,77,78. A previously developed semi-automated macro was adapted to this analysis 56. Briefly, after tracing the cell body using the freehand selection tool, the following shape descriptors were obtained: area (μm^2^), perimeter (μm), circularity (4π × (area/perimeter^2^)), aspect ratio (major axis/minor axis, a.u.), roundness (arbor area/area of a circle with same convex perimeter, a.u.) and solidity (area/convex cell area, a.u.). A circularity of 1.0 represents a perfect circle and 0.0 is indicative of an elongated cell shape. Similarly, values higher than a 1.0 aspect ratio correspond to more elongated cell shapes. A solidity of 1.0 reflects a less ramified, convex shape, whereas 0.0 solidity points to a porous cell shape that is more ramified. Moreover, values closer to 1.0 for roundness represent more circular cell shapes 30.

Next, the polygon tool was used to trace the endpoints of the microglial processes, creating a convex shape that served as a proxy for the cellular territory, described through the aforementioned shape descriptors. The convex shape was subsequently processed to produce an unsharp mask of the cell, using the “remove outliers”, “clear outside” and “despeckle” functions. When necessary, the unsharp mask was manually corrected using the brush function to represent the observed cell. Once polished, the mask was used to obtain the preceding shape descriptors. The same mask was processed with the “analyze particles” and “analyze skeleton” commands to calculate the number of branches, endpoints, average branch length, maximum branch length (μm) and longest shortest path (μm), increasing with cell ramification. The mask arbour was also converted into an outline and analyzed using the FracLac 79 plugin to obtain the fractal dimension (a.u.) and lacunarity (a.u.), as previously described 30. Fractal dimension and lacunarity are complementary measures 79, with higher values indicating a more complex organization of branching and more ramified morphological states 30. Albeit informative, morphological analyses can be challenging to extrapolate across research teams 61 and represent one level of complexity of microglial function that should be complemented 7.

### Scanning electron microscopy staining, processing and imaging

Double immunohistochemistry staining against Iba1 and IgG was performed in 3 sections (in both the ipsilateral and contralateral hemispheres per animal; n = 3 animals/time point) using a similar protocol as described above. A few steps in the protocol were adapted to improve tissue preservation at the nanometric scale 19. The quenching was performed with 0.3% H_2_O_2_ in PBS for 7 min; the blocking buffer and antibody incubation solutions contained 0.01% Triton X-100 and, after the DAB incubations, the sections were left in PB overnight at 4 °C.

The following day, sections were post-fixed flat using an osmium-thiocarbohydrazide-osmium protocol 30, in which samples were first incubated in 3% potassium ferrocyanide (cat# PFC232.250, BioShop, Ontario, Canada) diluted in PB and combined (1:1) with 4% aqueous osmium tetroxide (cat# 19170, Electron Microscopy Sciences) for 1 h at RT. After washing with ultrapure H_2_O, the sections were immersed in 1% thiocarbohydrazide (cat# 2231-57-4, Electron Microscopy Sciences) for 20 min and an additional layer of 2% osmium tetroxide was applied for 30 min, with both solutions diluted in ultrapure H_2_O at RT. This was followed by dehydration with ascending concentrations of ethanol (35%, 50%, 70%, 80%, 90%, 100%) and propylene oxide (cat# 110205, Millipore Sigma) for 5 min each at RT. Next, sections were embedded in Durcupan ACM resin (cat# 44611-44614, Millipore Sigma) overnight at RT. The following day, sections were polymerized with a thin layer of resin between two fluoropolymer sheets (ACLAR; cat# 50425-25, Electron Microscopy Sciences) placed for 72 h at 55 °C. The *CA1 LMol* was excised from the flat-embedded sections on ACLAR® sheets and glued to the top of resin blocks (2/hemisphere/animal, n = 3 animals/time point). Ultrathin sections (~ 75 nm) were generated with an ultramicrotome (ARTOS 3D ultramicrotome, Leica Biosystems, Ontario, Canada), collected on a silicon nitride chip, and glued onto specimen mounts for SEM. In each resin block, 6 levels (~ 5 μm apart) of ultrathin sections were collected. One ultrathin section was imaged per level using a Zeiss Crossbeam 350 Focused-Ion Beam SEM (Zeiss, Baden-Württemberg, Germany). Iba1^+^ microglial cell body were selected and the images exported as TIFF files with the Zeiss ATLAS Engine 5 software (Fibics, Ontario, Canada) at a resolution of 5 nm per pixel.

### Ultrastructural analyses

To quantify ultrastructural changes, we analyzed 151 microglial cell bodies (n = 11-13 microglial cell bodies from each ipsilateral and contralateral hemisphere, N = 3 animals/time point). This sample size was sufficient to obtain statistical power based on calculations using G*Power software 19,76 and is consistent with previous literature in the field, in which tissue is examined at both the light- and electron-microscopy levels 39,77,78. Images were analyzed using QuPath V0.4.3 80 and FIJI 73, adapted for use in previous work from our lab 39. Microglial cell bodies were identified based on DAB staining, their smaller cell bodies and nuclei than neighbouring astrocytes or neurons, a characteristic heterochromatin pattern, long stretches of ER cisternae, and the presence of inclusions (e.g., lysosomes and lipofuscin granules) dispersed in their cytoplasm 19,30,31. Microglial processes were identified based on their positive staining for Iba1, but were only analyzed qualitatively. Prior to analysis, a total of 3 trained and blinded observers separately agreed on the microglial identity of the selected cells.

Microglial cell body contacts with other cell bodies (i.e., astrocytes and neurons), myelinated axons, blood vessels, and synaptic elements (pre-synaptic axon terminals and post-synaptic spines) were quantified by an experimenter blinded to the experimental conditions 74,81,82. The ultrastructural criteria used to identify each structure are outlined below. Astrocytes and astrocytic endfeet had pale nuclei with a thin rim of heterochromatin and pale irregular cytoplasm, often containing intermediate filaments 83. Astrocytic endfeet were considered swollen when increased drastically in size, showing a clear cytoplasm enclosed by a plasma membrane 35. Neurons presented pale nuclei and cytoplasm, and direct contacts with pre-synaptic terminals 83. Myelinated axons were characterized by electron-dense sheaths and granular cytoplasm, often presenting mitochondria. Degraded myelin was recognized by ballooning, swelling or distancing between the well-defined myelin sheaths 83. Blood vessel area (major radius*minor radius*π) was recorded using the freehand tool after two orthogonal diameters were traced, each delimited by the basement membrane, which was identified as an electron-dense layer surrounding endothelial cells forming blood vessels. The distance between each microglia membrane (from either a cell body or process) and the closest basement membrane was also traced 19,30,31. Microglia were associated with a blood vessel when their distance from the vascular basement membrane was under 150 nm 19,30,31. Moreover, among the parenchyma, microglial contacts with erythrocytes, which are recognized by their shape, size, lack of a nucleus and mitochondria, and completely electron-dense cytoplasm, were counted. Microglial contacts with synaptic elements were categorized as contacts with axon terminals or dendritic spines 19,30,31. Pre-synaptic axon terminals showed a minimum of five synaptic vesicles and were usually in contact with post-synaptic spines displaying a visible post-synaptic density 83. Extracellular space pockets, essential for microglial motility and neuronal remodeling, were classified based on clear spaces without delineating membranes directly surrounding the microglia 39,82. Extracellular digestion refers to extracellular space pockets containing debris in the vicinity of a microglial cell body or process, often in proximity to degraded myelin (ballooning, swelling or distancing between the well-defined myelin sheaths) 35.

The intracellular ultrastructural state of microglia, e.g., ER/Golgi apparatus, lysosomes, lipofuscin, mitochondria, and phagosomes, were also characterized 19,30,31. ER/Golgi cisternae were identified by their long and narrow stretches. ER/Golgi apparatus dilation, associated with cellular stress, was identified when the distance between cisternal membranes was greater than 100 nm 17,19. Mitochondria (homeostatic) were found in the cytoplasm, presenting an electron-dense appearance, double membrane, numerous cristae, and a circular shape. Mitochondria bigger than 1 μm in length were established as elongated, a phenomenon typically observed in response to cellular stress 77. Moreover, when presenting with deteriorated outer membranes, vacuoles or degradation in their cristae membranes (electron-lucent pockets), mitochondria were classified as dystrophic 19,30,31. Events of proximity between mitochondria and ER/Golgi apparatus cisternae, associated with calcium transfer and lipid synthesis 78, were quantified. Moreover, endothelial, astrocytic endfeet and synaptic elements containing mitochondria were counted. Mitochondrial apposition to plasma membranes has been proposed to represent a component of purinergic signaling attracting microglial cell or process contacts 36. Primary lysosomes were identified by their circular and homogeneous contents (digestive enzymes) enclosed by a single membrane. Secondary lysosomes were darker, at least twice larger than primary lysosomes and often fused with phagosomes containing digested materials. Tertiary lysosomes were the largest, presenting residual materials, such as lipofuscin, large lipid bodies and phagosomes 19,30,31. Lipid bodies are sites for synthesis and storage of immune mediators, including inflammatory cytokines 79, and were characterized by their electron-dense circular shape and interior. Oval structures with electron-dense content and a unique fingerprint-like pattern were identified as lipofuscin. Phagosomes were defined by their ovoid or circular shape with a single membrane and electron-lucent interior. They were classified as empty (completely electron-lucent) or filled (electron-lucent with content) 19,30,31. Autophagosomes, part of an intracellular degradation pathway, were instead identified by the presence of elements inside circular, double-membrane vacuoles with a clear interior, resembling the cellular cytoplasm 19,80.

### Statistics

Microglial density, distribution, morphology and ultrastructural statistical analyses were conducted using the software GraphPad Prism (V 8.0.0 for MAC OS Ventura 13.4, GraphPad Software, California, USA). Normality of the data was assessed using a Shapiro-Wilk test. Mixed effects 2-way ANOVA with Šídák’s multiple comparison correction was used to compare the animal averages (n) for contrast intensity, optical density, microglial density and distribution, morphology, and ultrastructure between ipsilateral and contralateral hippocampi at the two examined time points 39. As this is an exploratory study, all post-hoc pairwise comparisons were done, though only analyses comparing ipsilateral *vs* contralateral and 1 h *vs* 24 h are reported in the text. Both nonparametric and parametric data were used for the 2-way ANOVA, considering the lack of a non-parametric alternative to this test. The frequency distribution of cell values (morphology and ultrastructure) was assessed using the Gaussian or sum of 2 Gaussian nonlinear regression models for modal and bimodal data, respectively. The Wilcoxon test was applied to the nonlinear models comparing ipsilateral *vs* contralateral* LMol* in each time point 74. When the number of recorded events within the cell dataset was not high enough to create the nonlinear model, we computed the presence *vs* absence of the variable across conditions. The conditional relative frequency of ultrastructural changes was measured using Fisher’s exact test. Spearman r correlations were computed for density and distribution, morphology and ultrastructural datasets separately, as described below. Mean differences were considered statistically significant when p < 0.05, with **** p < 0.0001, *** p < 0.001, ** p < 0.01, * p < 0.05. All reported values are presented as mean ± standard error of the mean (S.E.M).

### Correlation analyses

The ipsilateral *CA1* density and distribution (n = 3 animals/time point) dataset and the optical density values (IgG staining) were imported into the GraphPad Prism built-in correlation tool to compute relationships between measured features. The same approach was used to explore the ipsilateral relationships between the 30 morphological features analyzed and optical density (n = 3 animals/time point) (Table S5). In addition, contralateral and ipsilateral correlations between the microglial cell body distance to the closest vessel and vessel area to all microglial ultrastructural features were analyzed (n = 151 cells, N = 3 animals/time point) (Table S11).

### Principal component analyses

The Graph-Pad Prism built-in principal component analysis (PCA) was used to reduce dimensionality of the ipsilateral and contralateral morphology and ultrastructure datasets. Briefly, the variables were standardized to have a mean 0 and standard deviation of 1 to obtain a correlation matrix. The PC were selected based on a parallel analysis. Moreover, loadings and PC scores were visualized using scatterplots. Loadings and PC scores were used to understand the morphological and ultrastructural features that contributed to most of the variability in the ipsilateral compared to the contralateral *LMol* datasets.

## Supplementary Material

Supplementary materials and methods, figures and tables.

## Figures and Tables

**Figure 1:**
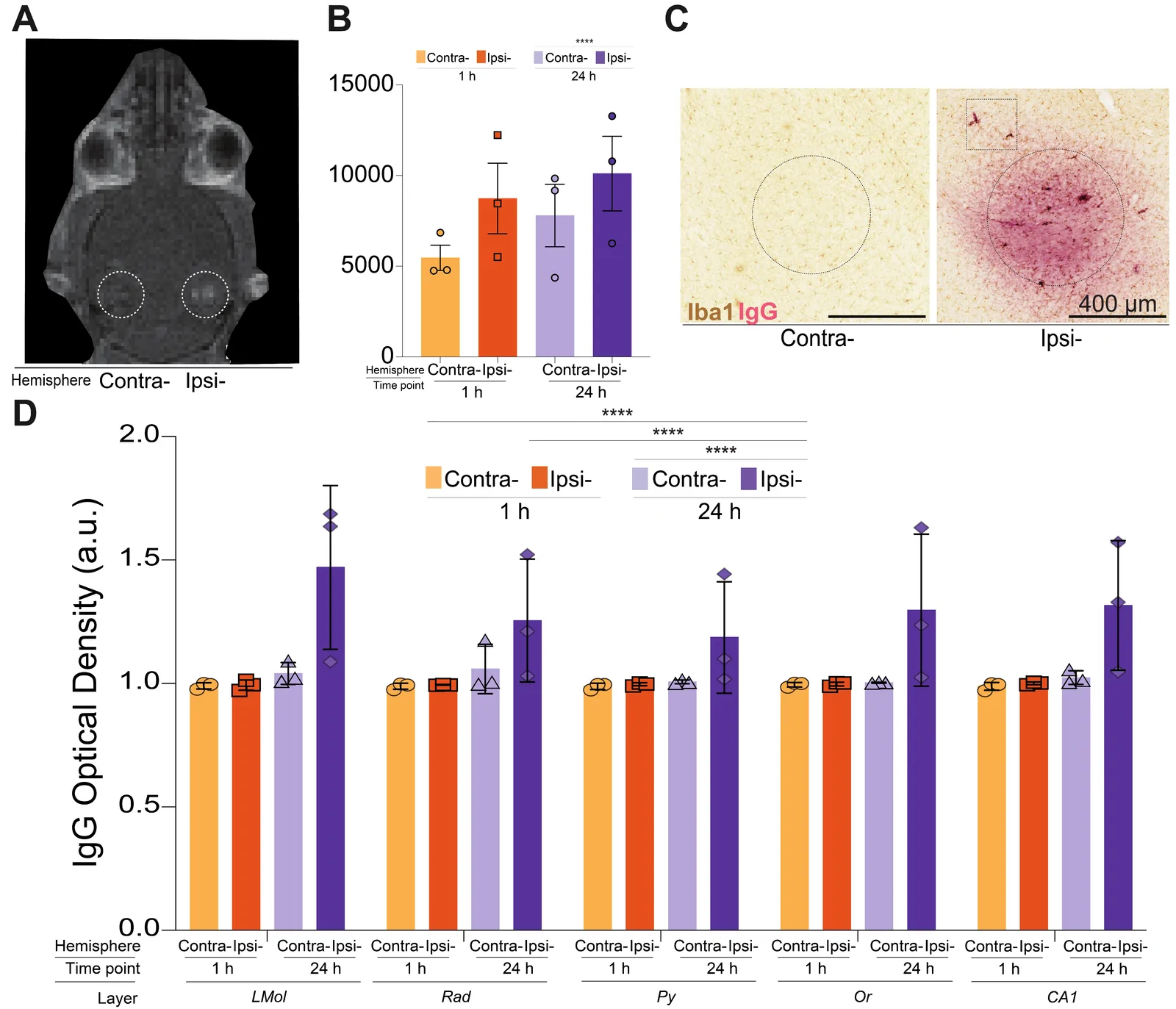
** FUS-BBB modulation increased hippocampal BBB permeability. A.** T1-weighted images confirmed increased blood-brain barrier (BBB) permeability indicated by gadolinium detection (dashed circle) in the ipsilateral (ipsi-) hippocampi parenchyma at 1 h and 24 h after FUS-BBB modulation. **B.** When quantified, the average voxel intensity of the gadolinium signal was comparable in the ipsilateral compared to the contralateral (contra-) hippocampi. The bar graph shows the mean, standard error of the mean, and individual data points (n = 3 animals/hemisphere/time point), analyzed with a mixed effects 2-way ANOVA. **C.** Representative images of ionized calcium-binding adapter molecule 1 (Iba1), a marker of microglia/macrophages, and immunoglobulin (Ig)G immunoperoxidase staining imaged in the *cornu ammonis 1 (CA1)* at 40x with a brightfield microscope. IgG was only detected in the ipsilateral parenchyma and was often circular (dashed circle), indicative of the area targeted by the FUS-BBB modulation. Scale bars 400 mm. **D.** IgG optical density was significantly increased across time (main effect of time not shown) with higher values at 24 h compared to 1 h and contralateral hippocampus, irrespective of layer (*LMol*,* Rad, Py*,* Or*,* CA1*). The bar graphs show the mean, standard error of the mean, and individual data points (n = 3 animals/hemisphere/time point), analyzed with a mixed-effects 2-way ANOVA and Šídák’s multiple comparison tests. * *p <* 0.05, ** *p <* 0.01, *** *p <* 0.001, **** *p* < 0.0001. a.u.: arbitrary unit, *LMol: stratum lacunosum-moleculare*, *Or*: *stratum oriens*, *Py*: *stratum pyramidale* and *Rad*: *stratum radiatum*.

**Figure 2:**
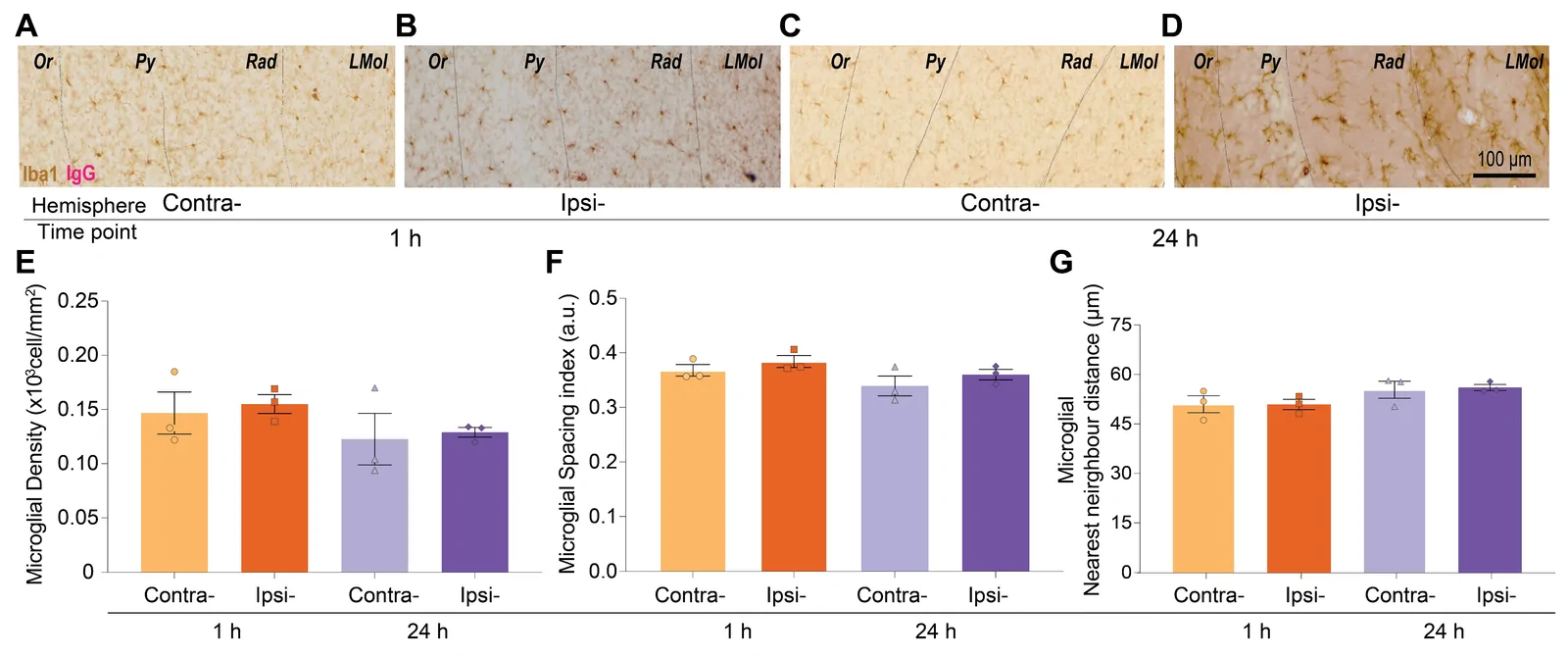
** Within one day of FUS-BBB modulation, there were no microglial density or distribution changes. A-D.** Representative brightfield microscopy images taken at 40x illustrate ionized calcium-binding adapter molecule 1 (Iba1) positive (^+^) cell and immunoglobulin (Ig)G^+^ parenchymal distribution along the examined *cornu ammonis 1 (CA1)* strata. Images were obtained in ipsilateral (ipsi-) and contralateral (contra-) hemispheres at 1 h and 24 h following double immunoperoxidase staining. Scale bar 100 μm. **E-G.** In the *CA1*, the microglial density and spacing index were not altered despite a main effect of time (not shown) in changing the nearest neighbour distance during the 24 h after FUS-BBB modulation (**G**). The bar graphs show the mean, standard error of the mean, and individual data points (n = 3 animals/hemisphere/time point). Statistical significance was assessed by a mixed-effects 2-way ANOVA with Šídák’s multiple comparison tests. a.u.: arbitrary unit, *LMol: stratum lacunosum-moleculare*, *Or*: *stratum oriens*, *Py*: *stratum pyramidale* and *Rad*: *stratum radiatum*.

**Figure 3:**
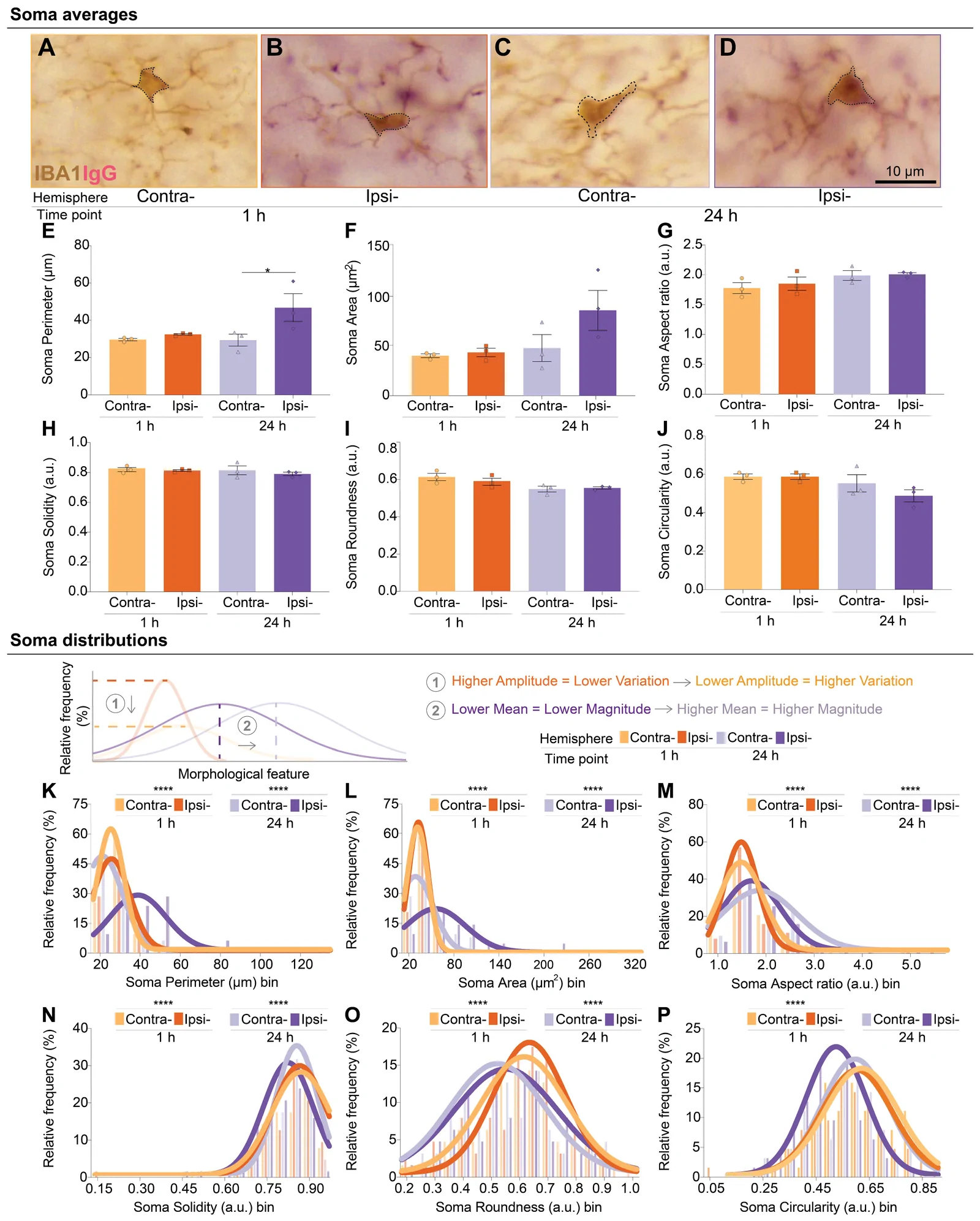
** The distribution of microglial soma shape descriptors shifted one day following FUS-BBB modulation. A-D.** Representative brightfield microscopy images taken at 40x illustrate mouse ionized calcium-binding adapter molecule 1 (Iba1) positive (^+^) cells with soma tracings (dashed line) across the contralateral (contra-) and ipsilateral (ipsi-) *lacunosum moleculare* (*LMol*) 1 h and 24 h following FUS-BBB. Scale bar 10 μm. **E–J.** The average soma perimeter (**E**) was significantly increased in the ipsilateral *vs* contralateral *LMol* at 24 h, according to a main effect of hemisphere, an interaction effect of hemisphere x time (both not shown) and post-hoc effect for contralateral *vs* ipsilateral at 24 h. The mean Iba1^+^ soma area (**F**), aspect ratio (**G**), solidity (**H**), roundness (**I**), and circularity (**J**) did not change in the ipsilateral *LMol* at 1 h or 24 h, compared to the contralateral controls. The bar graphs show the mean, standard error of the mean, and individual data points (n = 3 animals/hemisphere/time point) analyzed by a mixed-effects 2-way analysis of variance (ANOVA) with Šídák’s multiple comparison tests. **K–P.** According to nonlinear regression models, the relative distribution of Iba1^+^ soma perimeter (**K**), area (**L**), aspect ratio (**M**), solidity (**N**), and roundness (**O**) was significantly distinct in the ipsilateral compared to the contralateral *LMol* at both 1 h and 24 h, though only at 1 h soma circularity (**P**). Histograms show the relative frequency, and nonlinear regression was analyzed via Wilcoxon comparison tests. * *p* < 0.05, ** *p* < 0.01, *** *p* < 0.001, **** *p* < 0.0001. a.u.: arbitrary unit, IgG: immunoglobulin G.

**Figure 4:**
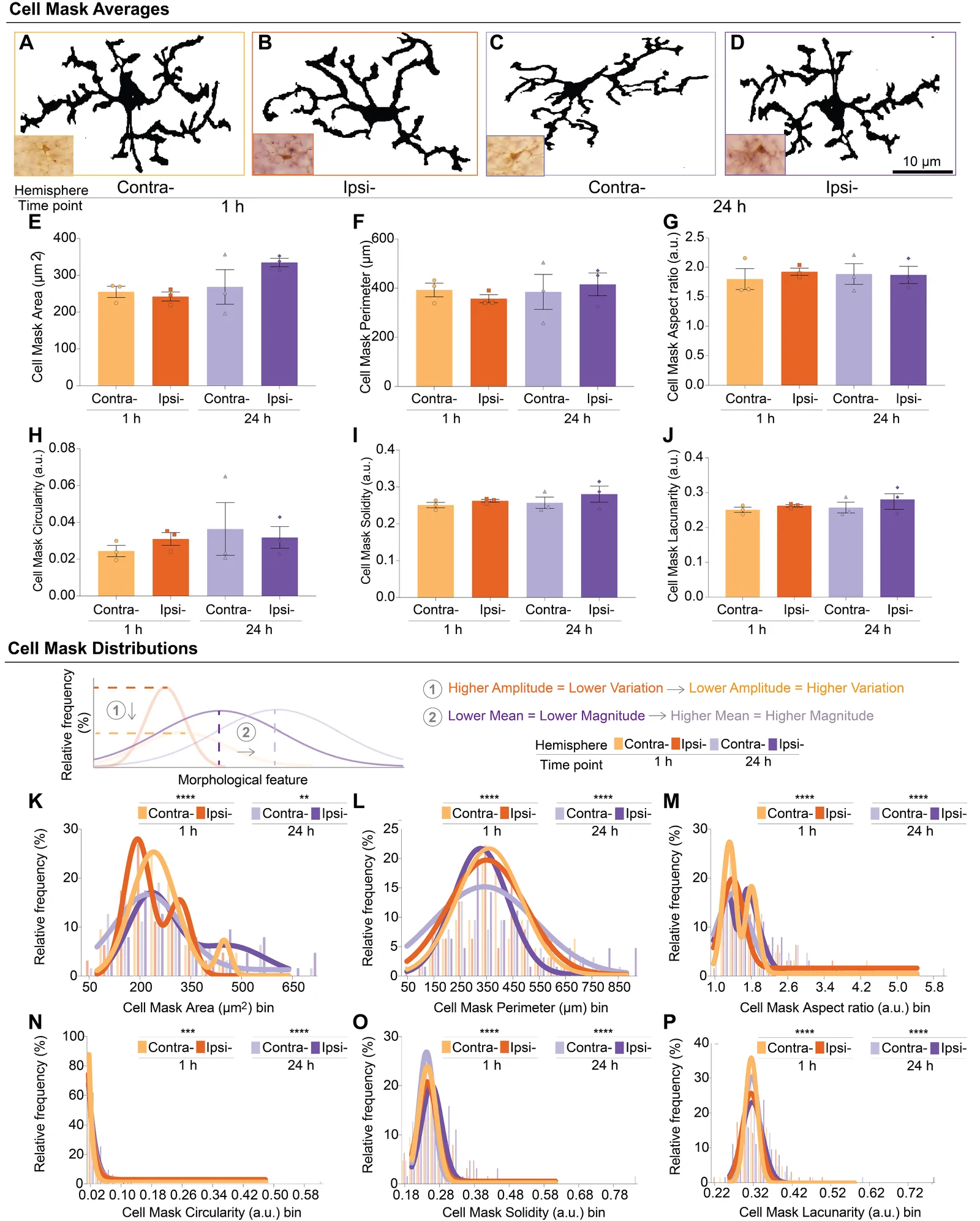
** The distribution of microglial processes changed within one day of FUS-BBB modulation. A–D**. Representative brightfield microscopy automated cell masks obtained from 40x magnification images illustrate ionized calcium-binding adapter molecule 1 (Iba1) positive (^+^) cells across the 1 h and 24 h contralateral (contra-) and ipsilateral (ipsi-) *lacunosum moleculare (LMol)* following FUS-BBB. Scale bar 10 μm. **E–J**. Iba1^+^ automated cell mask area (**E**), perimeter (**F**), aspect ratio (**G**), circularity (**H**), solidity (**I**), and lacunarity (**J**) averages did not change in the ipsilateral *LMol* at 1 h and 24 h, compared to the contralateral controls. Bar graphs show the mean, standard error of the mean and individual data points (n = 3 animals/hemisphere/time point) analyzed by a mixed-effects 2-way analysis of variance (ANOVA). **K–P**. However, according to nonlinear regression models, the relative distributions of automated cell mask area (**K**), perimeter (**L**), aspect rati**o (M), c**ircularity (**N**), solidity (**O**) and lacunarity** (P)** were significantly distinct in the ipsilateral compared to the contralateral control at both 1 h and 24 h. Histograms show the relative frequency and nonlinear regression analyzed via Wilcoxon comparison tests. * p < 0.05, ** p < 0.01, *** p < 0.001, **** *p* < 0.0001. a.u.: arbitrary unit.

**Figure 5:**
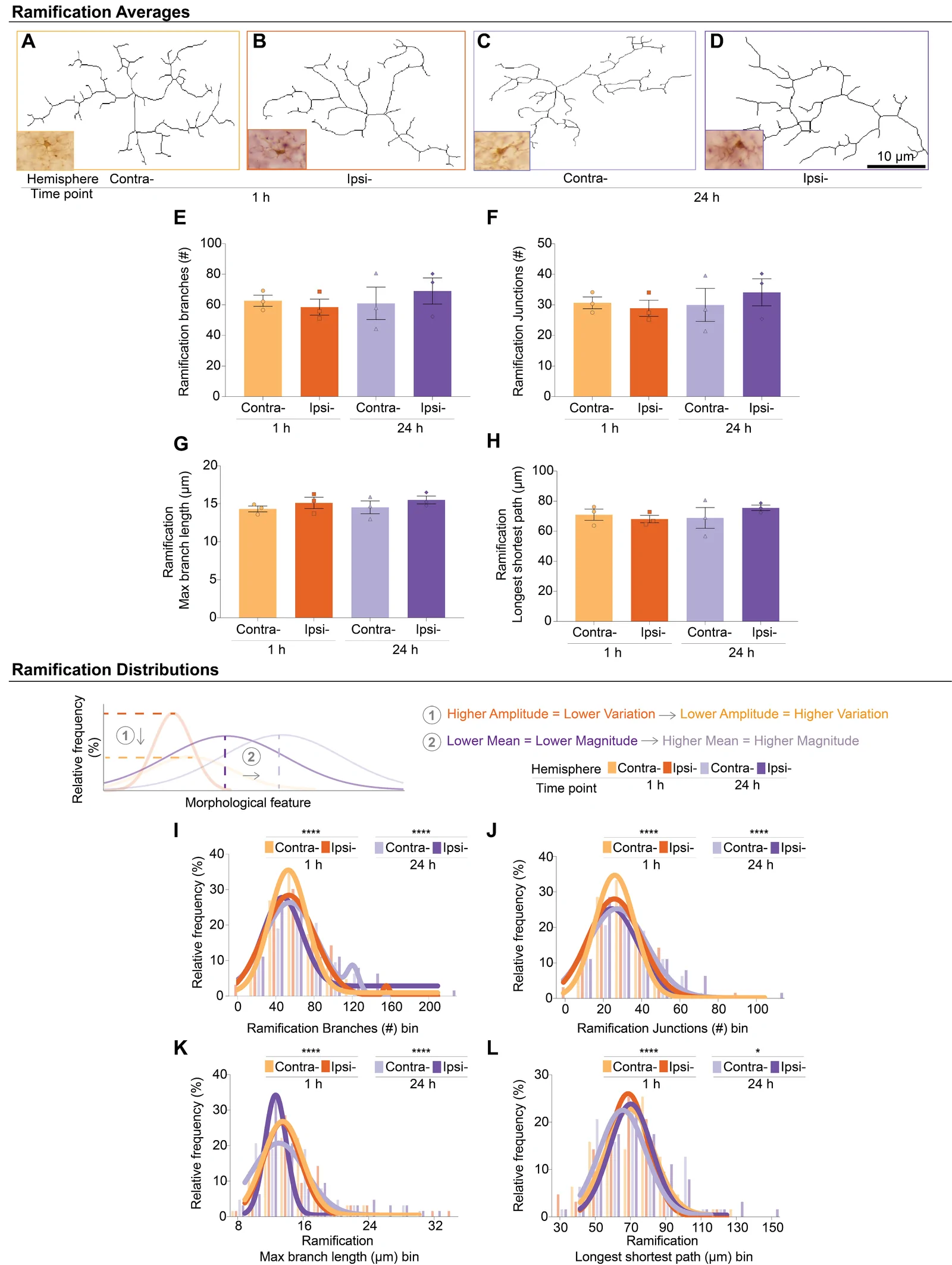
** The length and number of microglial processes fluctuated 1 h and 24 h following FUS-BBB modulation. A–D.** Representative brightfield microscopy arborizations obtained from 40x magnification images illustrate ionized calcium-binding adapter molecule 1 (Iba1) positive (^+^) cells across the 1 h and 24 h contralateral (contra-) and ipsilateral (ipsi-) *lacunosum moleculare (LMol)* following FUS-BBB. Scale bar 10 μm. **E–H.** The number of branches (**E**) and junctions (**F**), along with the maximum branch length (**G**) and longest shortest path (**H**) averages, did not change in the ipsilateral *LMol* at 1 h or 24 h, compared to the contralateral controls. The bar graphs show the mean, standard error of the mean, and individual data points (n = 3 animals/hemisphere/time point) analyzed by a mixed-effects 2-way analysis of variance (ANOVA).** I–L.** By contrast, according to nonlinear regression models, the relative distributions of branches (**I**) and junctions (**J**) number, as well as maximum branch length (**K**) and longest shortest path (L), were significantly distinct in the ipsilateral *LMol* compared to the contralateral control at both 1 h and 24 h. Histograms show the relative frequency and nonlinear regression analyzed via Wilcoxon comparison tests. * p < 0.05, ** p < 0.01, *** p < 0.001, **** *p* < 0.0001. a.u.: arbitrary unit.

**Figure 6:**
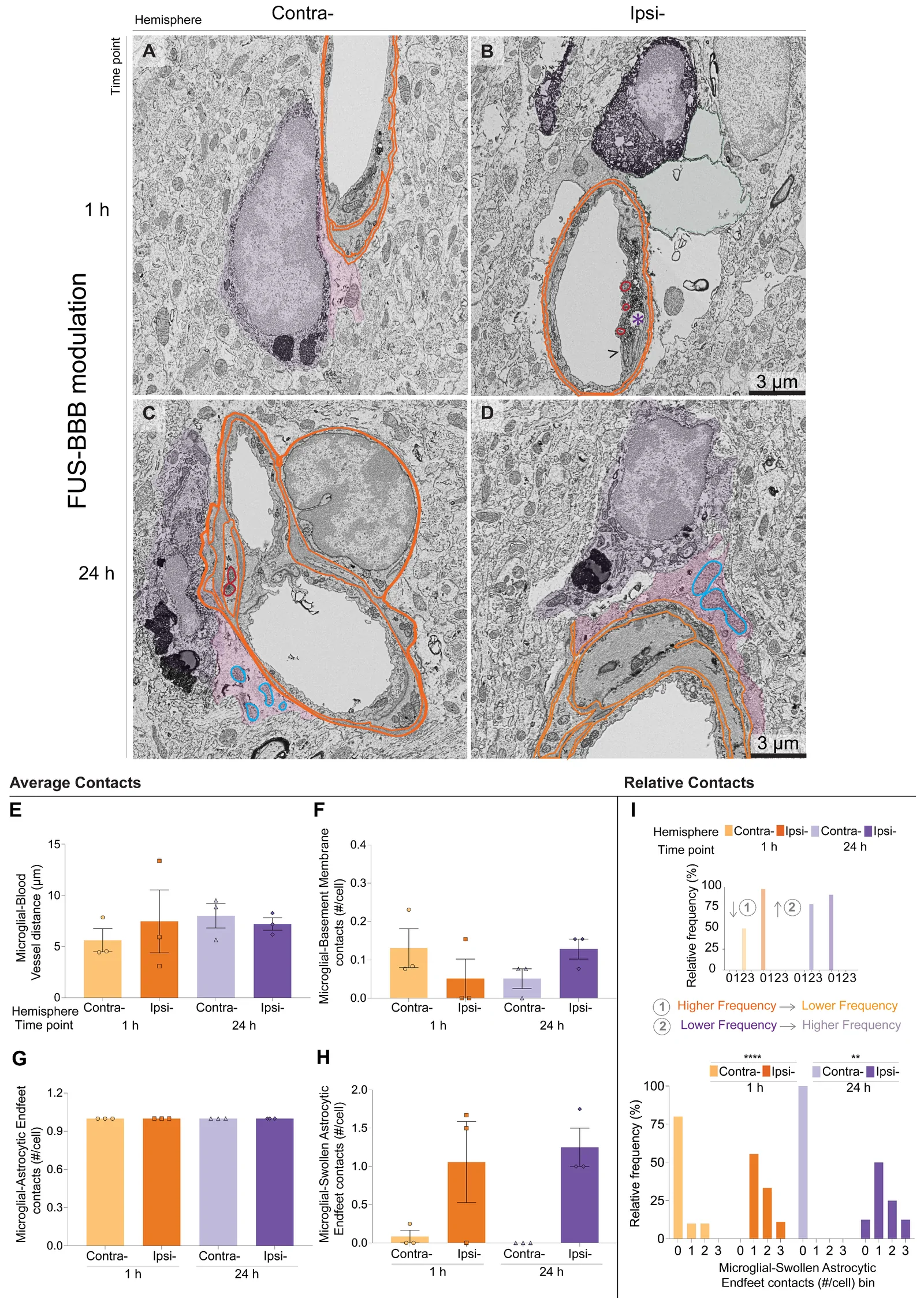
** FUS-BBB modulation increased the frequency of microglial contact with swollen astrocytic endfeet. A-D**. Representative scanning electron microscopy (SEM) images of ionized calcium-binding adapter molecule 1 (Iba1) positive (^+^) cell bodies (purple) and processes (purple asterisk), alongside blood vessels in the ipsilateral (ipsi-) and contralateral (contra-) *stratum lacunosum moleculare* (*LMol*) at 1 h and 24 h after FUS-BBB. Microglial contacts with endothelial cells and astrocytic endfeet contain mitochondria, pseudocolored in red and blue, respectively. Scale bar 3 μm. **E–H**. The average distance between microglia and blood vessels (**E**), the number of contacts between microglia and the basement membrane (**F,** orange), astrocytic endfeet (**G**, pink) and swollen astrocytic endfeet (**H**, green) did not differ between the ipsilateral and contralateral *LMol* of mice at 1 h and 24 h after FUS-BBB modulation, despite an interaction effect between hemisphere and time for basement membrane contacts and a hemisphere effect for swollen endfeet contacts (both not shown). The bar graphs show the mean, standard error of the mean, and individual data points (n = 3 animals/hemisphere/time point) analyzed by a mixed-effects 2-way analysis of variance (ANOVA).** I**. By contrast, the relative number of contacts between microglia and swollen astrocytic endfeet significantly increased in the ipsilateral compared to the contralateral *LMol* at 1 h and 24 h after FUS-BBB modulation. Relative frequency bar graphs analyzed via Fisher’s exact tests. *p < 0.05, **p < 0.01, ***p < 0.001.

**Figure 7:**
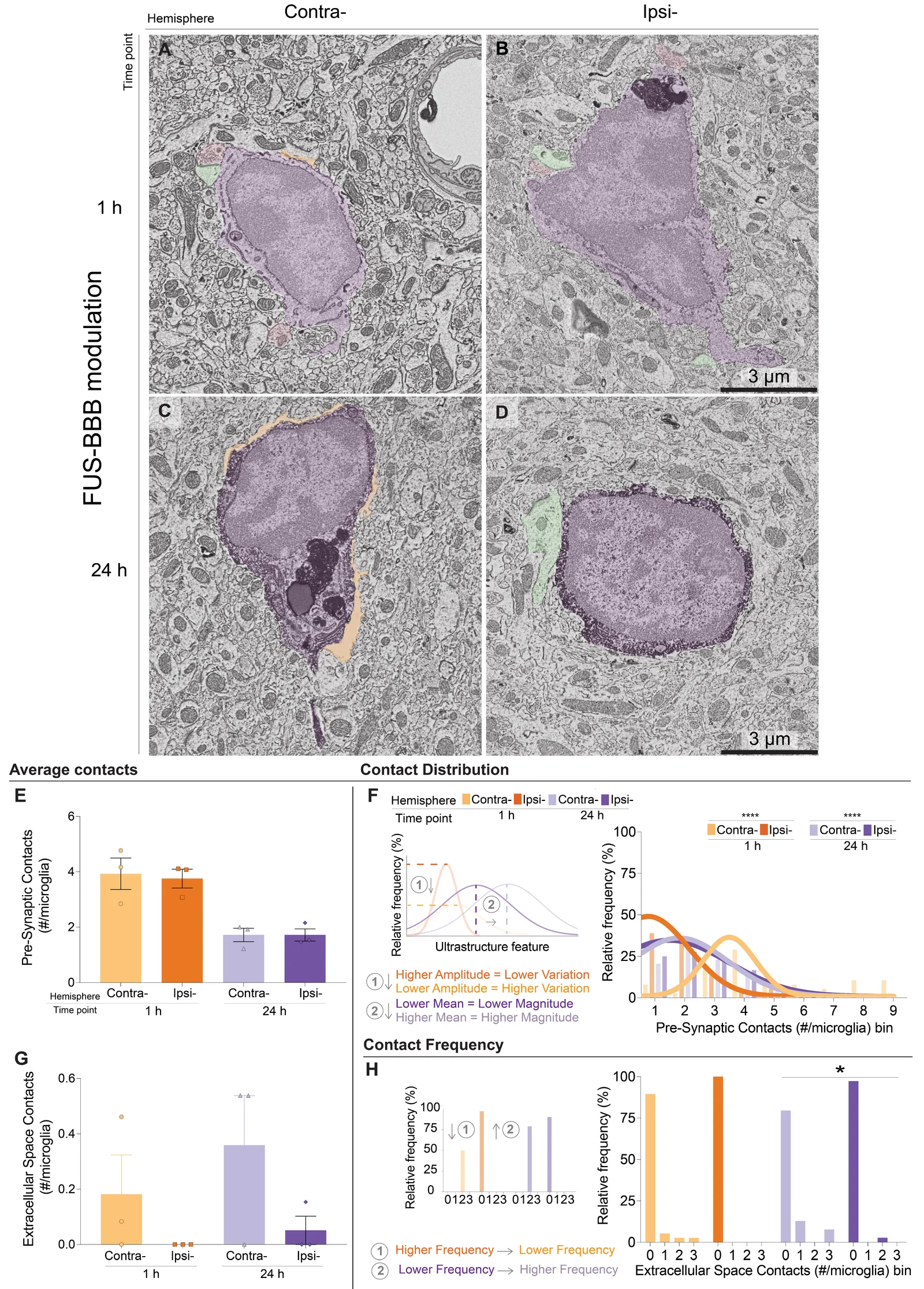
** Microglia interacted less frequently with pre-synaptic elements and extracellular space pockets following FUS-BBB modulation. A–D**. Representative scanning electron microscopy (SEM) images of ionized calcium-binding adapter molecule 1 (Iba1) positive (^+^) cells (purple) and blood vessels in the ipsilateral (ipsi-) and contralateral (contra-) *stratum lacunosum moleculare* (*LMol*) at 1 h and 24 h after FUS-BBB. Microglial contacts with pre-synaptic elements (**A, B,** pink), post-synaptic elements (**A**, **B, D,** green) and extracellular space pockets (**C**, beige). Scale bar 3 μm. **E**. The average number of contacts between microglia and pre-synaptic elements was significantly modulated by time (main effect not shown), suggesting a reduction in contacts during the 24 h after FUS-BBB modulation. The bar graph shows the mean, standard error of the mean, and individual data points (n = 3 animals/hemisphere/time point) analyzed by a mixed-effects 2-way analysis of variance (ANOVA) with Šídák’s multiple comparisons tests**. F**. According to nonlinear regression modeling, the relative distribution of ultrastructural interactions between microglia and pre-synaptic elements significantly differed between the ipsilateral and contralateral *LMol* at 1 h and 24 h after FUS-BBB modulation. Histograms show the relative frequency, and nonlinear regression analyzed via Wilcoxon comparison tests. **G**. The average number of ultrastructural interactions with extracellular space pockets did not differ for microglial cell bodies examined at 1 h or 24 h after FUS-BBB modulation in the ipsilateral *vs* contralateral *LMol*. **H**. However, at 24 h, the relative frequency of contacts with extracellular space per microglial cell body decreased in the ipsilateral *LMol* compared to the contralateral *LMol* at 24 h. Histograms show the relative frequency analyzed by Fisher’s exact tests. *p < 0.05, **p < 0.01, ***p < 0.001. #: number.

**Figure 8:**
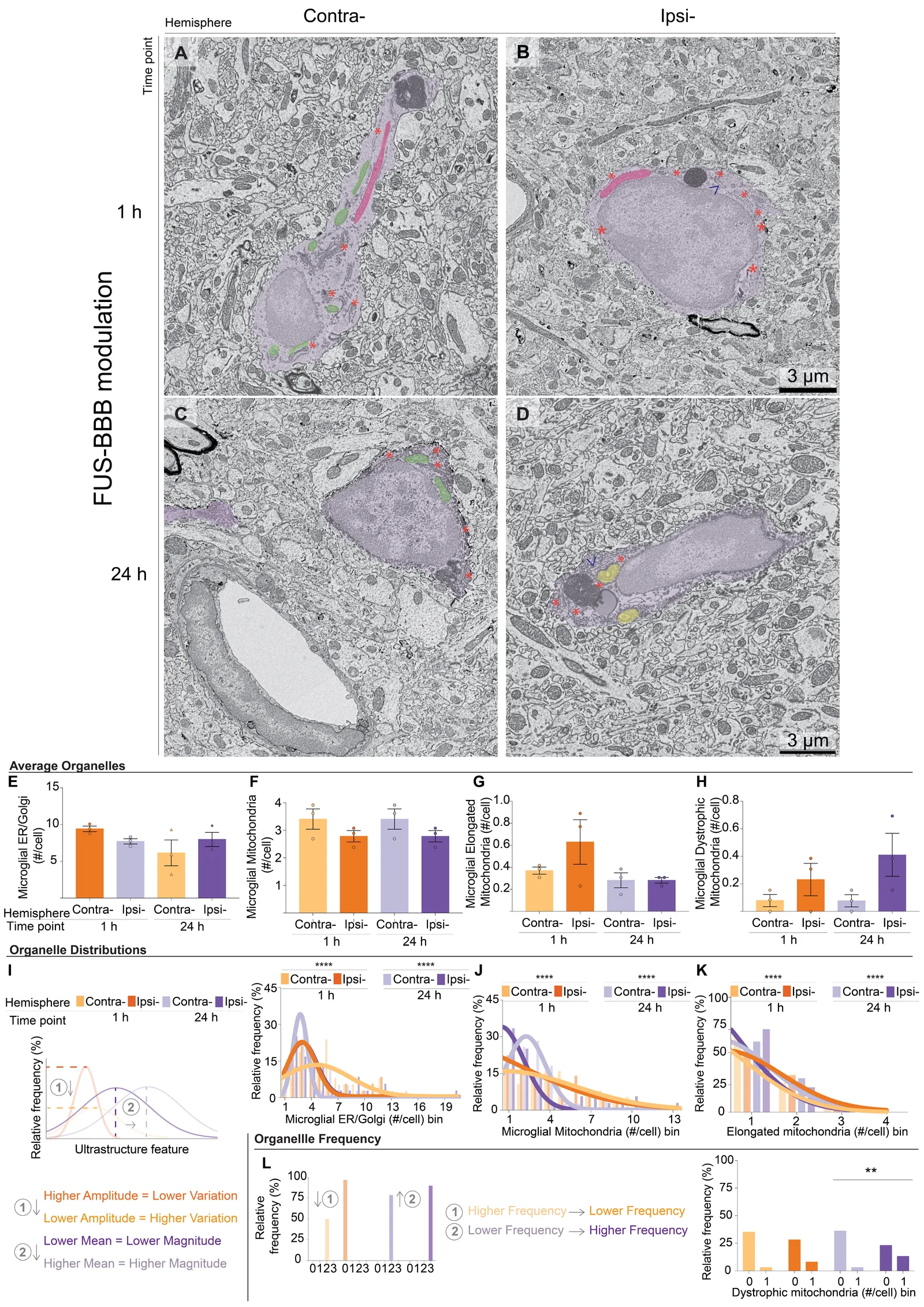
** Microglia altered their mitochondria and endoplasmic reticulum following FUS-BBB modulation. A–D.** Representative scanning electron microscopy (SEM) images of ionized calcium-binding adapter molecule 1 (Iba1) positive (^+^) cells (purple) and blood vessels in the ipsilateral (ipsi-) and contralateral (contra-) *stratum lacunosum moleculare* (*LMol*) at 1 h and 24 h after FUS-BBB. Pictures highlight microglial homeostatic mitochondria (green) and endoplasmic reticulum/Golgi apparatus (ER/Golgi, orange asterisk) cisternae (**A, C**), elongated mitochondria (**A, B**, pink), dilated ER/Golgi (**B, D**, blue arrowhead), and dystrophic mitochondria (**D**, orange). Scale bars: 3 μm. **E–H**. The average number of microglial ER/Golgi cisternae (**E**), mitochondria (**F**), elongated mitochondria (**G**) and dystrophic mitochondria (**H**) did not change at 1 h and 24 h after FUS-BBB modulation in the ipsilateral and contralateral *LMol,* despite a main effect of hemisphere for homeostatic and dystrophic mitochondria (both not shown). The bar graphs show the mean, standard error of the mean, and individual data points (n = 3 animals/hemisphere/timepoint) analyzed by a mixed-effects 2-way analysis of variance (ANOVA). **I-K**. According to the nonlinear regression models, the relative frequency of ER/Golgi (**I**), mitochondria (**J**) and elongated mitochondria (**K**) differed between the ipsilateral *vs* contralateral *LMol* at 1 h and 24 h. **L**. Histograms show the relative frequency and nonlinear regression analyzed via Wilcoxon comparison tests. The number of dystrophic mitochondria per cell was significantly different between ipsilateral and contralateral *LMol* at 24 h. Histograms show the relative frequency analyzed via Fisher’s exact tests. *p < 0.05, **p < 0.01, ***p < 0.001. #: number.

**Figure 9:**
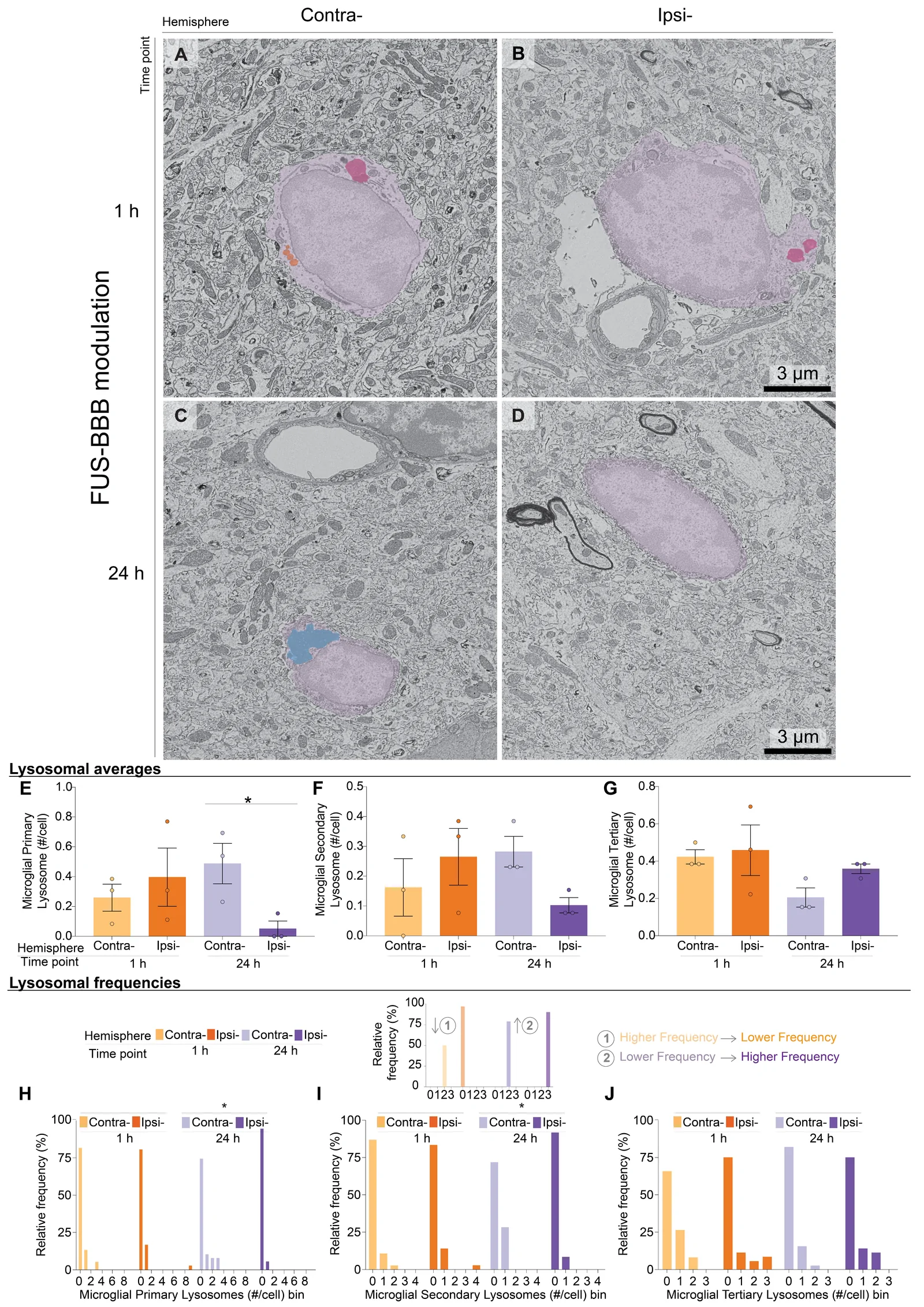
** Microglia had fewer primary lysosomes following FUS-BBB modulation. A–D.** Representative scanning electron microscopy (SEM) images of ionized calcium-binding adapter molecule 1 (Iba1) positive (^+^) cells (purple) and blood vessels in the ipsilateral (ipsi-) and contralateral (contra-) *stratum lacunosum moleculare* (*LMol*) at 1 h and 24 h after FUS-BBB. Pictures highlight primary (**A**, orange), secondary (**B**, pink) and tertiary (**C**, blue) lysosomes and microglial cell bodies without visible lysosomes (**D**). Scale bars: 3 μm. **E**. The average number of primary lysosomes was significantly decreased in microglial cell bodies of the ipsilateral *vs* contralateral *LMol* at 24 h after FUS-BBB, according to both an interaction effect between hemisphere and time (not shown) and post-hoc comparison.** F**. A significant interaction effect (not shown) that did not pass pairwise comparisons was observed for the average of secondary lysosomes between ipsilateral and contralateral *LMol*.** G**. The average number of tertiary lysosomes did not change between ipsilateral and contralateral *LMol* at 1 h and 24 h after FUS-BBB. The bar graphs show the mean, standard error of the mean, and individual data points (n = 3 animals/hemisphere/time point) analyzed by a mixed-effects 2-way analysis of variance (ANOVA) with Šídák’s multiple comparisons tests. **H–J**. The relative frequency of primary (**H**) and secondary lysosomes (**I**) was also significantly different between ipsilateral and contralateral *LMol* at 24 h after FUS-BBB, with no changes in tertiary lysosomes (J). Histograms show relative frequencies analyzed via Fisher’s exact tests. *p < 0.05, **p < 0.01, ***p < 0.001.

**Table 1:** Summary of mean and amplitude nonlinear regression models in ipsilateral *versus* contralateral microglia.

Ipsilateral *vs* Contralateral		1 h		24 h	
		Higher	Lower	Higher	Lower
Soma	Mean	area, perimeter, roundness, aspect ratio	solidity, circularity	area, perimeter, roundness	solidity, aspect ratio
	Amplitude	area, roundness, solidity, aspect ratio	perimeter, circularity	aspect ratio	area, perimeter, roundness, solidity
Territory	Mean	aspect ratio, circularity, roundness	area, perimeter	area, aspect ratio	circularity, roundness
	Amplitude	circularity, perimeter	area, aspect ratio, roundness	circularity	area, aspect ratio, roundness
Mask	Mean		area, perimeter, roundness, solidity, lacunarity, fractal dimension	area, solidity, lacunarity, fractal dimension	perimeter, roundness
	Amplitude	area	perimeter, roundness, solidity, lacunarity, fractal dimension	area, perimeter, roundness, fractal dimension	solidity, lacunarity
Arbor	Mean	branches	junctions, longest shortest path, maximum branch length	longest shortest path	branches, junctions, maximum branch length
	Amplitude	longest shortest path	branches, junctions, maximum branch length	junctions, longest shortest path, maximum branch length	branches

## Abbreviations

BBB: blood-brain barrier
FUS: transcranial-focused ultrasound
MRI: magnetic resonance imaging
Ig: immunoglobulin
CNS: central nervous system
ER: endoplasmic reticulum
Iba1: ionized calcium-binding adaptor molecule 1
SEM: scanning electron microscopy
*CA*: *cornu ammonis*
*LMol*: *stratum lacunosum*-*moleculare*
*Rad*: *stratum radiatum*
*Py*: *stratum pyramidale*
*Or*: *stratum oriens*
NND: nearest neighbour distance
PBS: phosphate-buffered saline
PB: phosphate-buffered
RT: room temperature
ROI: regions of interest
a.u.: arbitrary unit
S.E.M: standard error of the mean
PCA: principal component analysis
h: hour
ipsi-: ipsilateral
contra-: contralateral
^+^: positive
ANOVA: analysis of variance
