# Supplementary material for: Early micro- and nanoscale microglial responses to blood–brain barrier modulation by transcranial focused ultrasound
Source: Theranostics. 2026 Sep 18;16(16):9322–45. doi: 10.7150/thno.131051 (PMC13684825; doi:10.7150/thno.131051)
Supplement: Supplementary file 1 [file thnov16p9322s1.pdf]

Early micro- and nanoscale microglial responses to blood–brain barrier modulation by transcranial focused ultrasound Elisa Gonçalves de Andrade[1,2], Jared VanderZwaag[1,2], Rikke Hahn Kofoed[3,4,5], Katherine Picard[2,6], Keelin Henderson Pekarik[2], Micaël Carrier[2,6], Fernando González Ibáñez[2,6], Mohammadparsa Khakpour[2], Kullervo Hynynen[7,8,9], Isabelle Aubert[5,10,*], and Marie-Ève Tremblay[2,11,12,*]

[1]Neuroscience Graduate Program, University of Victoria, Victoria, BC, Canada
[2]School of Medical Sciences, Faculty of Health, University of Victoria, Victoria, BC, Canada
[3]Department of Neurosurgery, Department of Clinical Medicine, Aarhus University, Aarhus, Denmark
[4]Center for Experimental Neuroscience-CENSE, Department of Neurosurgery, Aarhus University Hospital, Aarhus, Denmark
[5]Biological Sciences, Hurvitz Brain Sciences Research Program, Sunnybrook Research Institute, Toronto, ON, Canada
[6]Axe neurosciences, Centre de recherche du CHU de Québec, Université Laval, Québec City, QC, Canada
[7]Temerty Chair in Focused Ultrasound Research, Sunnybrook Health Sciences Centre and University of Toronto, Toronto, ON, Canada
[8]Professor, Department of Medical Biophysics, University of Toronto, Toronto, ON, Canada
[9]Cross Appointed Professor, Institute of Biomedical Engineering, University of Toronto, Toronto, ON, Canada
[10]Department of Laboratory Medicine and Pathobiology, Temerty Faculty of Medicine, University of Toronto, Toronto, ON, Canada
[11]Department of Biochemistry and Molecular Biology, University of British Columbia, Vancouver, BC, Canada
[12]Centre for Advanced Materials and Related Technology (CAMTEC) and Institute on Aging and Lifelong Health (IALH), University of Victoria, Victoria, BC, Canada
*Co-correspondence:
Marie-Ève Tremblay: evetremblay@uvic.ca
Isabelle Aubert: isabelle.aubert@utoronto.ca; isabelle.aubert@sunnybrook.ca

Supplementary Materials

Supplementary Methods

The nonlinear regression models were characterized based on their reported amplitude and mean (Table S5, 10). A higher curve amplitude indicates a narrower distribution of values; therefore, less variability in that morphological feature across cells. A higher curve mean reflects a shift toward larger values of the feature across the population. We observed that microglia in the ipsilateral hemisphere deviated to larger and rounder somas with porous shapes, based on higher mean area, perimeter, roundness, but lower solidity at 1 h and 24 h following FUS-BBB (Figure 3K-P). In parallel, at 1 h there was a more homogeneous distribution of microglial soma area and compactness, based on the higher amplitude of soma area, roundness, solidity, and aspect ratio; although the inverse was true for soma perimeter and circularity amplitudes, which were lower, therefore, there was a larger spread of the distribution. At 24 h, the relative distribution of all soma shape descriptors became more heterogeneous; that is, there was lower amplitude in the ipsilateral compared to the contralateral *LMol,* except for the aspect ratio (Figure 3K-P).

Regarding the processes, microglial cells appeared to adopt smaller processes, given the lower mean area and perimeter, and more regular process shapes, as per the lower mean roundness, solidity, lacunarity, fractal dimension, of the regression curves in the ipsilateral *LMol* at 1 h. At 24 h, the process masks shifted back to larger, indicated by a higher mean area, more compact, as per a lower mean perimeter and roundness, and irregular shapes, due to higher mean solidity, lacunarity and fractal dimension, in the ipsilateral compared to *LMol* regressions (Table S5). Correspondingly, there was a more heterogeneous relative distribution of all process descriptors at 1 h, that is, lower amplitude, except for the area in the ipsilateral *vs* contralateral *LMol* (Table S5). The opposite was observed at 24 h, whereby there was a homogenous relative distribution of process mask size, indicated by higher area and perimeter amplitude, and shape complexity, referred to by higher fractal dimension and roundness amplitude; although porosity, represented by lower solidity and lacunarity amplitudes, remained heterogeneous (Table S5).

In the ultrastructural analysis, comparison of means of the nonlinear regression models suggested that the relative distribution of microglial contacts with pre-synaptic elements reduced at both time points, indicated by lower means in the ipsilateral compared to the contralateral *LMol*, resulting in a more homogeneous distribution of contacts at 1 h and the reverse effect at 24 h, represented by higher and

lower amplitudes, respectively (Table S10). Moreover, at 1 h in the ipsilateral *LMol,* the relative distribution of microglial cell bodies with higher metabolic demands increased, identified by elevated ER/Golgi and elongated mitochondria means, but decreased homeostatic mitochondria means, compared to the contralateral regressions. At 24 h, microglial ER/Golgi means were still higher, but microglial mitochondria and elongated mitochondria means decreased in the ipsilateral compared to the contralateral regressions. Such shifts resulted in an increasingly ipsilateral heterogeneous ER/Golgi distribution across microglial cell bodies at 1 h and 24 h, revealed by higher and lower amplitudes, respectively, with the reverse effect observed for homeostatic and elongated mitochondria, which showed lower amplitudes (Table S10).

Supplementary Figures

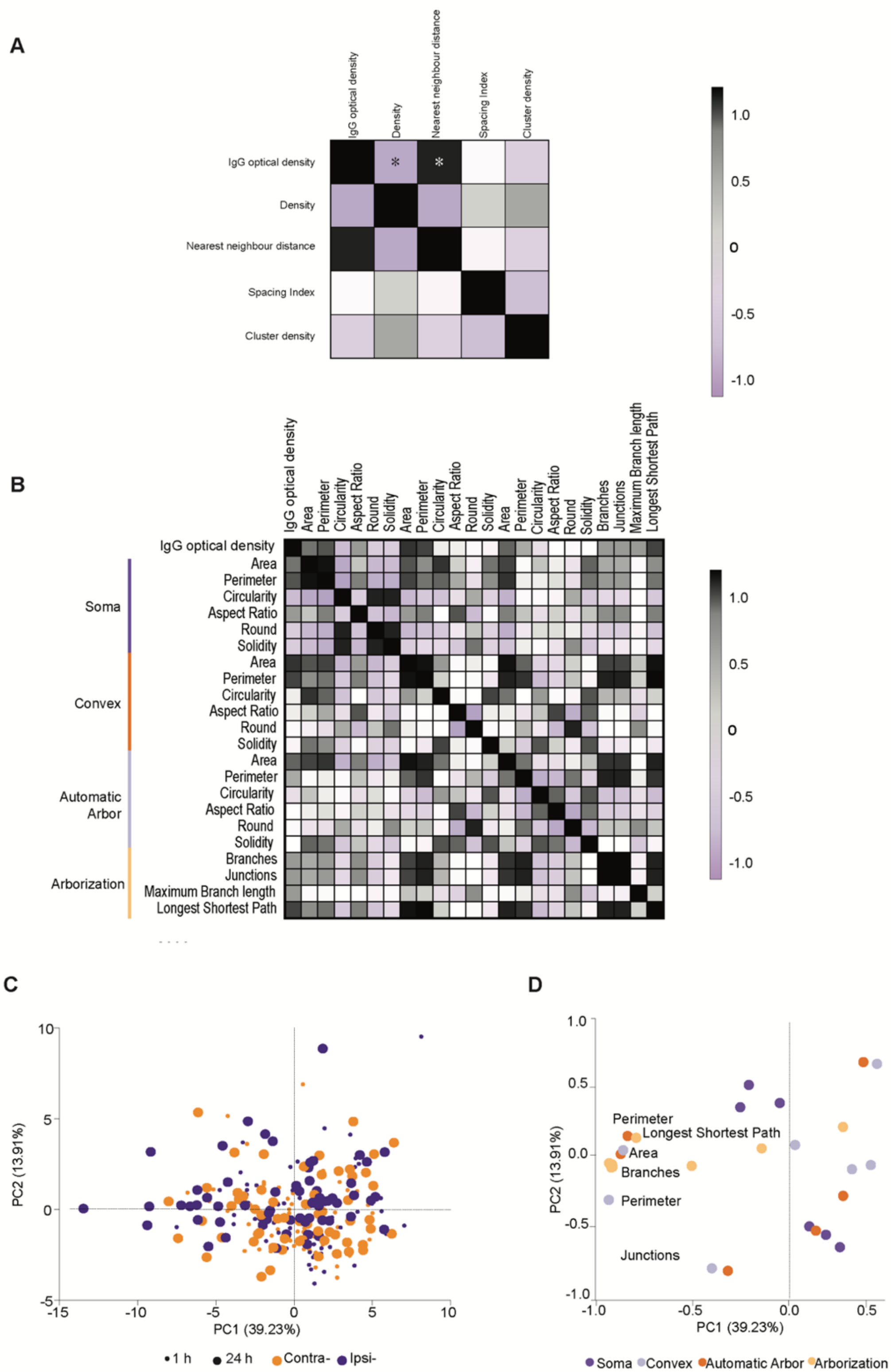

Figure S1. Changes in microglial density and distribution correlated with IgG immunostaining intensity at 1 h and 24 h FUS-BBB modulation. A. Optical density was significantly and negatively correlated with microglial density, with the reverse relationship observed for microglial nearest neighbour distance (NND) in the ipsilateral (ipsi-) *lacunosum moleculare* (*LMol*) at 1 h and 24 h following FUS-BBB modulation. **B.** Optical density did not correlate with any ipsilateral adaptations in morphology. Correlation matrices of ipsilateral features analyzed by Spearman r. **C.** Scores for the first and second principal component (PC1/PC2) of morphological features for all the cells analyzed, colored according to the *LMol* hemisphere (orange: contralateral, purple: ipsilateral) and time point (small icon: 1 h, big icon: 24 h). A higher variability in parameters is present at 24 h, particularly in the ipsilateral region group. **D.** Loading plot of morphology dataset, depicting the correlation between the morphological features (light orange: arborization, dark orange: convex shape, light purple: automatic arbor, dark purple: soma) and PC1/PC2, suggesting that variability in arborization descriptors contribute to overall differences between ipsilateral and contralateral *LMol* ionized calcium-binding adapter molecule 1 (Iba1) positive (+) cells.

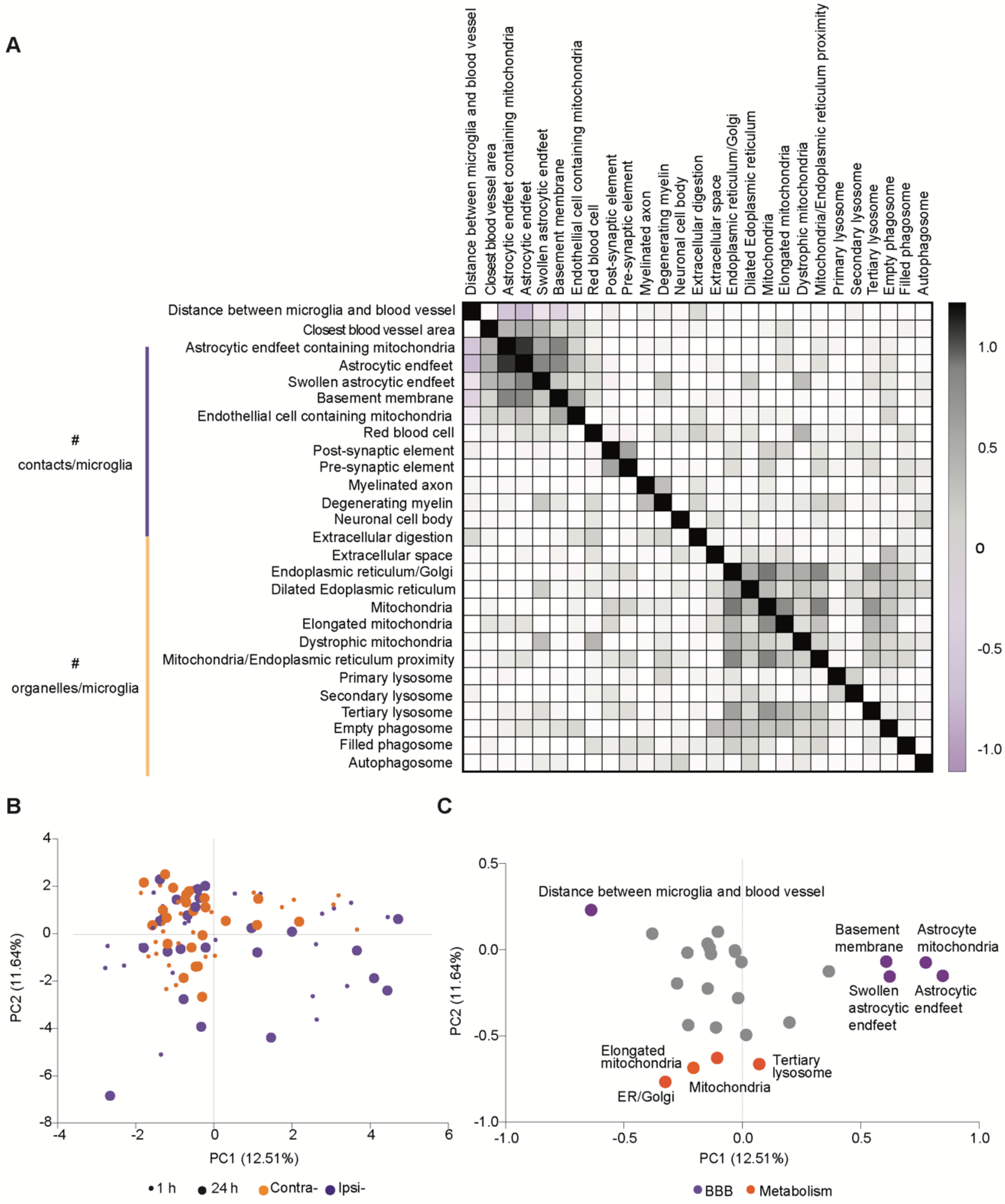

Figure S2. The microglial ultrastructure varied with FUS-BBB modulation. A. The microglial cell body distance from blood vessels was negatively correlated, while it was positively correlated with changes in microglial cell body contacts with astrocytic endfeet, swollen astrocytic endfeet, basement membrane and endothelial cells containing mitochondria in the ipsilateral (ipsi-) *stratum lacunosum moleculare* (*LMol)* at 1 h and 24 h following FUS-BBB modulation. Correlation matrix of ipsilateral features analyzed by Spearman r. **B**. Scores for the first and

second principal components 1 and 2 (PC1/PC2) for the microglial ultrastructural features analyzed, colored according to the *LMol* hemisphere (orange: contralateral, purple: ipsilateral) and time point (small icon: 1 h, big icon: 24 h). A higher variability in parameters is present at 24 h, particularly in the ipsilateral *LMol*. **C**. Loading plot of ultrastructural dataset, depicting the correlation between the ultrastructural features (orange: microglial cellular stress, purple: microglial contacts with the blood-brain barrier or BBB) and PC1/PC2, suggesting that most adaptations between the ipsilateral and contralateral *LMol* ionized calcium-binding adapter molecule 1 (Iba1) positive (+) cells in the microglial ultrastructure scanning electron microscopy dataset arise from microglial cell body interactions with the BBB and cellular stress.

Supplementary Tables

Table S1. Increased BBB permeability descriptive statistics.

p values are given after 2-way ANOVA of contra- and ipsi- average contrast intensity and of optical density absolute values in each *CA1* stratum (with Šídák's multiple comparisons test). For each dataset, SW results, mean and S.E.M of n = 3 animals/timepoint are given. ANOVA: analysis of variance, BBB: blood-brain barrier, Contra- or C: contralateral, Ipsi- or I: ipsilateral, SW: Shapiro-Wilk normality test, N: no, Y: yes, *p: p* value, f: f value, df: degrees of freedom and S.E.M.: standard error of the mean.

	Contra-			Ipsi-	
SW	Mean	S.E.M	SW	Mean	S.E.M
Average contrast intensity					
Y	6629	1165	Y	9421	688.9
Optical density					
Y	0.9904	0.0013	Y	0.9966	0.0012

Average contrast intensity 2-way ANOVA		
df = 4	*p*	f
Time	0.4273	0.7789
Hemisphere	0.0720	5.906
TimexHemisphere	0.6998	0.1718

Optical density 2-way ANOVA		
df = 30	*p*	f
LayerxTime	0.9408	0.43
Layer	0.7686	0.45
**Time**	**<0.0001**	17.49

Optical Density - Šídák's multiple comparisons	
Comparison	*p*
C1xI1	0.8933
C1xC24	0.9993
**C1xI24**	**<0.0001**
I1xC24	0.9355
**I1xI24**	**<0.0001**
**C24xI24**	**<0.0001**

Table S2. Microglial density and distribution descriptive statistics.

p values are given after mixed effects 2-way ANOVA with Šídák's multiple comparisons test comparing averages per animal for each *CA1* strata across the two timepoints (1 h and 24 h). For each data, SW results, mean and S.E.M of n = 3 animals/timepoint/hemisphere are given. ANOVA: analysis of variance, BBB: blood-brain barrier, *CA1*: *cornus ammonis* 1, *LMol*: *stratum lacunosum moleculare*, *Or*: *stratum oriens*, *Py*: *stratum pyramidal* and *Rad*: *stratum radiatum* C or Contra-: contra-, I or Ipsi-: ipsi-, SW: Shapiro-Wilk normality test, N: no, Y: yes, p: p value, f: f value, t: t distribution, df: degrees of freedom, T: timepoint, H: hemisphere, h: hours, S.E.M. standard error of the mean and NND: nearest neighbour distance.

	Contra-			Ipsi-		
	SW	Mean	S.E.M	SW	Mean	S.E.M
	Density (cells/μm^2)					
LMol		0.00010	0.00000		0.00010	0.00000
Rad		0.00010	0.00000		0.00020	0.00000
Py	Y	0.00010	0.00000	Y	0.00020	0.00000
Or		0.00010	0.00000		0.00010	0.00000
CA1		0.00010	0.00000		0.00020	0.00000
	Cluster density (cells/μm^2)					
LMol	N	0.00	0.00	N	0.00	0.00
CA1		0.00	0.00	Y	0.00	0.00
	NND (a.u.)					
LMol		53.20	2.20		53.50	2.57
Rad		53.30	1.41		50.40	2.45
Py	Y	73.70	2.54	Y	69.00	0.68
Or		56.50	2.67		54.90	4.21
CA1		68.00	4.38		61.90	8.59
	Spacing index (a.u.)					
LMol		0.35	0.01		0.37	0.01
Rad		0.41	0.03		0.39	0.01
Py	Y	0.53	0.03	Y	0.49	0.08
Or		0.40	0.02		0.45	0.04
CA1		0.76	0.06		0.64	0.12

Mixed effects 2-way ANOVA			
Layer	Main effect	p	f
Density (µm2)			
LMol	T	0.13	3.6
	H	0.22	2.1
	TxH	0.35	1.1
Rad	T	0.28	1.3
	H	0.28	1.3
	TxH	0.25	1.6
Py	T	0.35	1.0
	H	0.67	0.2
	TxH	0.89	0.0
Or	T	0.22	2.1
	H	0.78	0.1
	TxH	0.42	0.8
CA1	T	0.16	2.4
	H	0.66	0.2
	TxH	0.95	0.0
Cluster density (µm2)			
LMol	T	0.50	0.5
	H	0.13	2.9
	TxH	0.13	2.9
CA1	T	0.82	0.1
	H	0.19	2.1
	TxH	0.36	0.9
NND (a.u.)			
LMol	T	0.71	0.2
	H	0.13	3.6
	TxH	*0.06*	6.8
Rad	T	0.18	2.7
	H	0.47	0.6
	TxH	0.49	0.6
Py	T	0.12	3.0
	H	0.44	0.7
	TxH	0.59	0.3

Mixed effects 2-way ANOVA			
Layer	Main effect	p	f
Or	T	0.81	0.1
	H	0.72	0.1
	TxH	0.89	0.0
CA1	**T**	**0.05**	**5.5**
	H	0.89	0.0
	TxH	0.86	0.0
LMol	T	0.31	1.3
	H	0.58	0.4
	TxH	0.43	0.8
Rad	T	0.18	2.6
	H	0.10	4.7
	TxH	0.55	0.4
Py	T	0.61	2.6
	H	0.67	4.7
	TxH	0.32	0.4
Or	T	0.39	0.8
	H	0.53	0.4
	TxH	0.76	0.1
CA1	T	0.07	4.2
	H	0.19	2.1
	TxH	0.87	0.0

Table S3. Microglia density and distribution correlation with BBB permeability descriptive statistics.

p values are given after Spearman r correlation between contrast enhancement and density, NND, spacing index, cluster density and optical density in the ipsi- *LMol*. For each data, SW results, mean, S.E.M, r and p of n = 3 animals/timepoint/hemisphere are given. NND: nearest neighbour distance, SW: Shapiro-Wilk normality test, N: no, Y: yes, p: p value, r: r coefficient and S.E.M. standard error of the mean.

				Density		NND		Spacing Index		Cluster density	
	SW	Mean	S.E.M	r	p	r	p	r	p	r	p
Density	Y	0.00	0.00	1.00		-0.94	0.02	0.20	0.71	0.38	0.47
NND		53.46	1.41	-0.94	0.02	1.00		-0.14	0.80	-0.49	0.33
Spacing Index		0.37	0.01	0.20	0.71	-0.14	0.80	1.00		-0.70	0.14
Cluster Density		0.00	0.00	0.38	0.47	-0.49	0.33	-0.70	0.14	1.00	
Optical Density		1.17	0.07	-0.94	0.02	0.89	0.03	-0.09	0.92	-0.52	0.30

Table S4. Microglial morphology descriptive statistics.

p values are given after a mixed-effects 2-way ANOVA with Šídák's multiple comparisons test comparing averages per animal across the ipsi- and contra- *LMol* across the two timepoints (1 and 24 h). For each data, SW results, mean and S.E.M of n = 3 animals/timepoint are given. ANOVA: analysis of variance, a.u.: arbitrary unit, #: number, *LMol*: *stratum lacunosum moleculare*, C or Contra-: contra-, I or Ipsi-: ipsi-, SW: Shapiro-Wilk normality test, *: N too small for normality assessment, p: p value, f: f value, t: t distribution, df: degrees of freedom, T: timepoint, H: hemisphere, h: hours and S.E.M. standard error of the mean.

	Contra-		Ipsi-		Mixed effects 2-way ANOVA		
Parameter	Mean	S.E.M	Mean	S.E.M		f	p
Soma							
Area (μm^2)	43.31	3.79	63.72	20.89	T	3.53	0.13
					H	3.35	0.14
					TxH	2.35	0.20
Aspect Ratio (a.u.)	1.88	0.11	1.93	0.08	T	4.76	0.06
					H	0.30	0.60
					TxH	0.11	0.75
**Perimeter (a.u.)**	29.46	0.13	39.58	7.15	T	1.82	0.25
					**H**	**15.43**	**0.02**
					**TxH**	**8.01**	**0.05**
**Roundness (a.u.)**	0.58	0.03	0.58	0.02	**T**	**11.17**	**0.01**
					H	0.11	0.75
					TxH	0.48	0.51
Circularity (a.u.)	0.57	0.02	0.54	0.05	T	3.06	0.16
					H	4.63	0.10
					TxH	4.63	w0.10
Solidity (a.u.)	0.82	0.00	0.80	0.01	T	0.39	0.56
					H	1.68	0.26
					TxH	0.57	0.49
Convex							
Area (μm^2)	1084.00	24.79	1138.00	151.90	T	1.86	0.21
					H	0.18	0.69
					TxH	0.96	0.36
Aspect Ratio (a.u.)	1.69	0.01	1.67	0.01	T	0.00	0.99
					H	0.07	0.81
					TxH	0.07	0.81
Perimeter (a.u.)	144.20	1.13	145.80	6.65	T	0.34	0.58
					H	0.03	0.87
					TxH	0.67	0.44
Roundness (a.u.)	0.67	0.01	0.65	0.02	T	1.08	0.36
					H	1.48	0.29
					TxH	4.11	0.11
Circularity (a.u.)	0.64	0.01	0.64	0.00	T	0.07	0.79
					H	0.02	0.88
					TxH	0.01	0.91
Solidity	0.89	0.01	0.90	0.01	T	4.48	0.07

(a.u.)					H	1.62	0.24
					TxH	0.02	0.88
Automated cell mask							
Area (μm^2)	261.60	6.73	288.50	46.17	T	3.76	0.12
					H	1.17	0.34
					TxH	2.52	0.19
Aspect Ratio (a.u.)	1.84	0.04	1.90	0.03	T	0.01	0.91
					H	0.14	0.72
					TxH	0.22	0.65
Roundness (a.u.)	0.60	0.00	0.59	0.00	T	0.00	0.99
					H	0.22	0.65
					TxH	0.01	0.92
Fractal dimension (a.u.)	1.39	0.01	1.38	0.01	T	0.00	0.99
					H	0.05	0.83
					TxH	0.56	0.48
Lacunarity (a.u.)	0.25	0.00	0.27	0.01	T	0.76	0.41
					H	1.56	0.25
					TxH	0.17	0.69
Circularity (a.u.)	0.03	0.01	0.03	0.00	T	0.63	0.45
					H	0.01	0.91
					TxH	0.47	0.51
Perimeter (µm)	388.60	3.95	386.20	29.25	T	0.31	0.59
					H	0.00	0.96
					TxH	0.53	0.49
Solidity (a.u.)	0.25	0.00	0.27	0.01	T	0.76	0.41
					H	1.56	0.25
					TxH	0.17	0.69
Arborization							
Branches (#)	61.81	0.84	63.78	5.29	T	0.30	0.61
					H	0.08	0.79
					TxH	0.78	0.43
Junctions (#)	30.33	0.34	31.49	2.60	T	0.30	0.61
					H	0.11	0.76
					TxH	0.68	0.46
Longest Shortest Path (µm)	69.91	1.09	71.80	3.72	T	0.39	0.55
					H	0.20	0.67
					TxH	1.30	0.29
Max branch length (µm)	14.43	0.10	15.32	0.19	T	0.20	0.66
					H	1.88	0.21
					TxH	0.02	0.90

Šidák's multiple comparisons test

	1 h *vs* 24 h				Contra- *vs* Ipsi-			
Parameter	H	p	t	df	T	p	t	df
Soma								
Perimeter (a.u.)	C	1.00	0.05		1	0.73	0.78	4
	I	0.08	2.47		24	**0.02**	4.78	

Table S5. Morphological Nonlinear regression parameters.

p values are given after paired Wilcoxon test of Contra- *vs* Ipsi- nonlinear regression curve fits. These were modeled after the relative distribution of each parameter in the two *LMol* (contra- and ipsi-) nested into the two timepoints (1 h and 24 h). For each data, Amplitude, Mean and p value of n = 3 animals/timepoint/hemisphere are given. a.u.: arbitrary unit, #: number, p: p value, *LMol*: *stratum lacunosum moleculare*, Contra-: contra-, Ipsi-: ipsi- and h: hours.

Nonlinear regression		1 h		24 h	
		Contra-	Ipsi-	Contra-	Ipsi-
Soma					
Area (µm²)	Amplitude	60.20	62.57	36.40	20.91
	Mean	37.09	38.20	34.36	59.99
	p	<0.0001		<0.0001	
Aspect Ratio (a.u.)	Amplitude	47.24	58.09	32.00	37.24
	Mean	1.55	1.55	1.86	1.72
	p	<0.0001		<0.0001	
Circularity (a.u.)	Amplitude	13.82	13.59	15.02	16.62
	Mean	0.59	0.57	0.57	0.49
	p	<0.0001		0.58	
Perimeter (µm)	Amplitude	61.38	46.52	47.87	28.05
	Mean	28.17	28.36	24.25	40.68
	p	<0.0001		<0.0001	
Roundness (a.u.)	Amplitude	13.17	14.80	12.38	11.77
	Mean	0.62	0.63	0.53	0.55
	p	<0.0001		<0.0001	
Solidity (a.u.)	Amplitude	21.81	23.14	27.40	23.78
	Mean	0.85	0.85	0.84	0.81
	p	<0.0001		<0.0001	
Convex arbor					
Area (µm²)	Amplitude	18.97	17.54	17.89	16.03
	Mean	988.40	912.80	969.20	1083.00
	p	<0.0001		0.00	
Aspect Ratio (a.u.)	Amplitude	20.40	18.19	22.24	20.15
	Mean	1.49	1.51	1.42	1.52
	p	<0.0001		<0.0001	
Circularity (a.u.)	Amplitude	14.45	14.76	17.06	17.90
	Mean	0.63	0.64	0.70	0.67
	p	<0.0001		<0.0001	
Perimeter (µm)	Amplitude	18.97	20.54	21.53	21.59
	Mean	140.10	137.00	138.30	145.30
	p	0.00		0.36	
Roundness (a.u.)	Amplitude	5.28	5.12	5.39	5.25
	Mean	0.18	0.18	0.20	0.17
	p	<0.0001		<0.0001	
Solidity (a.u.)	Amplitude	11.46	9.73	10.92	13.77
	Mean	0.89	0.98	0.95	0.93

	p	0.73		0.20	
Automatic arbor					
Aspect ratio (a.u.)	Amplitude	711.70	18.35	16.86	22.36
	Mean	1.65	1.47	1.49	1.61
	p	<0.0001		<0.0001	
Circularity (a.u.)	Amplitude	-33.45	-325.9	-45.64	-16.89
	Mean	0.39	0.04	0.39	0.07
	p	0.0003		<0.0001	
Perimeter (µm)	Amplitude	15.09	13.70	10.51	15.08
	Mean	367.00	359.20	349.10	334.70
	p	<0.0001		<0.0001	
Roundness (a.u.)	Amplitude	11.55	9.31	9.34	10.71
	Mean	0.63	0.60	0.62	0.58
	p	0.00		0.00	
Solidity (a.u.)	Amplitude	20.36	17.41	22.63	16.83
	Mean	0.25	0.25	0.25	0.27
	p	<0.0001		<0.0001	
Area (μm^2)	Amplitude	24.02	26.54	14.62	15.69
	Mean	241.80	187.30	223.00	231.80
	p	<0.0001		0.00	
Fractal Dimension (a.u.)	Amplitude	17.00	13.00	10.70	11.36
	Mean	1.40	1.38	1.38	1.39
	p	0.02		0.00	
Lacunarity (a.u.)	Amplitude	26.83	19.28	22.77	17.35
	Mean	0.32	0.32	0.32	0.32
	p	<0.0001		<0.0001	
Arborization					
Branches (#)	Amplitude	35.04	28.32	25.84	24.95
	Mean	56.75	57.23	59.33	53.89
	p	<0.0001		<0.0001	
Junctions (#)	Amplitude	34.47	27.79	25.04	25.12
	Mean	28.04	27.86	29.37	26.37
	p	<0.0001		<0.0001	
Longest Shortest Path (µm)	Amplitude	20.87	24.23	20.65	21.86
	Mean	70.54	69.25	64.74	71.46
	p	<0.0001		<0.0001	
Max Branch Length (µm)	Amplitude	26.37	26.04	20.26	33.79
	Mean	13.65	13.54	13.07	12.60
	p	<0.0001		<0.0001	

Table S6. Morphology correlation with BBB permeability.

p values are given after Spearman r correlation between optical density and morphology parameters in the ipsi- *LMol*. For each data, r and p of n = 3 animals/timepoint are given. p: p value, r: r coefficient.

	Optical density	
Parameters	p	r
Soma Area	0.24	0.60
Soma Perimeter	0.14	0.71
Soma Circularity	0.14	-0.71
Soma Aspect Ratio	0.42	0.43
Soma Roundness	0.30	-0.54
Soma Solidity	0.30	-0.54
Convex Area	0.06	0.83
Convex Perimeter	0.10	0.77
Convex Circularity	0.92	0.09
Convex Aspect Ratio	0.92	0.09
Convex Roundness	0.92	-0.09
Convex Solidity	0.80	-0.14
Automatic Arbor Area	0.18	0.66
Automatic Arbor Perimeter	0.50	0.37
Automatic Arbor Circularity	0.50	-0.37
Automatic Arbor Aspect Ratio	1.00	-0.03
Automatic Arbor Roundness	0.92	-0.09
Automatic Arbor Solidity	1.00	-0.03
Arborization Branches	0.42	0.43
Arborization Junctions	0.42	0.43
Maximum Branch length	0.42	0.43
Arborization Longest Shortest Path	0.10	0.77

Table S7. Microglia morphology principal component analysis.

For each PC, eigenvalue, proportion of variance, cumulative proportion of variance and loadings for each morphology parameter of n = 21 cells, N = 3 animals/timepoint are given. PC: principal components.

PC summary	PC1	PC2	PC3	PC4	PC5	PC6
Eigenvalue	11.77	4.172	3.04	1.951	1.887	1.556
Proportion of variance	39.23%	13.91%	10.13%	6.50%	6.29%	5.19%
Cumulative proportion of variance	39.23%	53.13%	63.27%	69.77%	76.06%	81.24%
Loadings						
Soma Area	-0.29	0.44	-0.49	0.18	-0.02	0.09
Soma Perimeter	-0.25	0.60	-0.65	0.04	-0.17	0.05
Soma Circularity	0.22	-0.56	0.60	0.26	0.01	-0.08
Soma Aspect Ratio	-0.09	0.47	-0.34	-0.26	0.62	-0.03
Soma Round	0.06	-0.41	0.32	0.27	-0.63	0.02
Soma Solidity	0.15	-0.47	0.59	0.22	0.24	-0.03
Convex Area	-0.92	0.10	0.02	0.02	-0.13	-0.22
Convex Perimeter	-0.88	0.23	0.21	-0.15	-0.15	0.04
Convex Circularity	0.10	-0.44	-0.48	0.34	0.18	-0.58
Convex Aspect Ratio	0.35	0.76	0.44	0.23	0.02	-0.08
Convex Round	-0.36	-0.73	-0.44	-0.27	-0.07	0.13
Convex Solidity	0.24	-0.19	-0.42	0.46	0.08	-0.66
Automatic Arbor Area	-0.90	0.13	-0.07	0.24	-0.12	0.11
Automatic Arbor Perimeter	-0.97	0.02	0.11	-0.01	-0.06	-0.10
Automatic Arbor Circularity	0.38	0.03	-0.20	0.46	-0.20	0.47
Automatic Arbor Aspect Ratio	0.42	0.75	0.36	0.24	0.04	-0.14
Automatic Arbor Round	-0.44	-0.71	-0.35	-0.27	-0.11	0.19
Automatic Arbor Solidity	0.29	0.00	-0.33	0.69	-0.05	0.46
Arborization Branches	-0.97	0.02	0.06	0.13	0.08	0.00
Arborization Junctions	-0.96	0.02	0.06	0.13	0.08	0.00
Maximum Branch length	-0.18	0.14	-0.02	-0.21	-0.59	-0.31
Arborization Longest Shortest Path	-0.84	0.22	0.19	0.01	-0.14	-0.13
Automatic Arbor Fractal Dimension	-0.76	-0.22	0.07	0.20	0.13	0.09
Automatic Arbor Lacunarity	-0.01	0.17	0.27	-0.30	0.00	0.13

Table S8. Microglial ultrastructure descriptive statistics.

p values are given after a mixed-effects 2-way ANOVA with Šídák's multiple comparisons test comparing averages per animal across the ipsi- and contra- Lmol across the two timepoints (1 h and 24 h). For each data, SW results, mean and S.E.M of n = 3 animals/timepoint are given. ANOVA: analysis of variance#: number, Lmol: *stratum lacunosum moleculare*, C or Contra- contra-, I or Ipsi-: ipsi-, SW: Shapiro-Wilk normality test, *: N too small for normality assessment, p: p value, f: f value, t: t distribution, df: degrees of freedom, T: timepoint, H: hemisphere, h: hours and S.E.M. standard error of the mean.

		Contra-		Ipsi-		Mixed effects 2-way ANOVA		
Parameter	unit	Mean	S.E.M	Mean	S.E.M		f	p
BBB contacts								
Astrocytic endfeet containing mitochondria		0.17	0.12	0.24	0.04	T	0.65	0.45
						H	0.55	0.48
						TxH	2.48	0.15
Astrocytic endfeet		0.24	0.13	0.51	0.08	T	1.36	0.31
						H	2.67	0.18
						TxH	0.11	0.76
Swollen astrocytic endfeet	#/cell	0.04	0.04	1.15	0.10	T	0.03	0.87
						**H**	**16.48**	**0.02**
						TxH	0.26	0.64
Endothelial cell containing mitochondria		0.05	0.05	0.08	0.03	T	0.53	0.51
						H	1.00	0.37
						TxH	1.00	0.37
Basement membrane		0.09	0.04	0.09	0.04	T	0.00	0.98
						H	0.00	0.96
						**TxH**	**12.31**	**0.02**
Microglial blood vessel distance	µm	6.82	1.20	7.34	0.13	T	0.36	0.56
						H	0.09	0.77
						TxH	0.56	0.47
Density of microglia associated with vessels	#/µm^2	0.17	0.07	0.24	0.02	T	0.97	0.38
						H	1.09	0.36
						TxH	0.62	0.47
Red blood cells contacts	#/cell	0.00	0.00	0.05	0.05	T	1.00	0.37
						H	1.00	0.37
						TxH	1.00	0.37
Synaptic plasticity contact								
Synapse containing mitochondria contact	#/cell	0.78	0.44	0.79	0.35	T	9.68	0.04
						H	0.01	0.94
						TxH	0.24	0.65
Post-synaptic element		0.84	0.17	0.97	0.30	T	3.41	0.14
						H	0.35	0.59
						TxH	0.35	0.59

Pre-synaptic element		2.82	1.11	2.74	1.02	**T**	**33.06**	**0.00**
						H	0.05	0.82
						TxH	0.05	0.82
Satellite		0.09	0.01	0.11	0.01	T	0.01	0.91
						H	0.09	0.77
						TxH	0.09	0.77
Myelinated axon		0.13	0.03	0.20	0.06	T	1.16	0.31
						H	0.74	0.41
						TxH	0.19	0.68
Degenerating myelin		0.07	0.01	0.08	0.03	T	0.56	0.47
						H	0.06	0.82
						TxH	0.08	0.79
Extracellular digestion		0.17	0.02	0.27	0.07	T	0.75	0.41
						H	2.87	0.13
						TxH	1.90	0.21
Extracellular space		0.27	0.09	0.03	0.03	T	0.95	0.36
						H	4.35	0.07
						TxH	0.29	0.61
Proximity to dark cells		0.16	0.00	0.05	0.00	T	0.00	0.97
						H	1.75	0.22
						TxH	0.00	0.97
Intracellular organelles								
ER/Golgi		7.79	1.63	7.85	0.13	T	1.75	0.26
						H	0.00	0.95
						TxH	3.66	0.13
Dilated ER		0.87	0.00	0.89	0.37	T	2.24	0.17
						H	0.00	0.95
						TxH	2.24	0.17
Mitochondria/ER		0.97	0.38	1.03	0.02	T	1.59	0.28
						H	0.10	0.77
						TxH	4.38	0.10
Mitochondria		3.41	0.00	2.78	0.00	T	0.00	>0.99
						**H**	**17.32**	**0.01**
						TxH	0.00	>0.99
Elongated mitochondria	#/cell	0.33	0.04	0.46	0.17	T	3.18	0.15
						H	1.94	0.24
						TxH	1.94	0.24
Dystrophic mitochondria		0.08	0.00	0.32	0.09	T	0.75	0.41
						H	5.59	0.05
						TxH	0.78	0.40
Primary lysosome		0.37	0.11	0.22	0.17	T	0.12	0.74
						H	3.45	0.14
						**TxH**	**12.72**	**0.02**
Secondary lysosome		0.22	0.06	0.18	0.08	T	0.05	0.83
						H	0.64	0.47
						**TxH**	**8.64**	**0.04**
Tertiary lysosome		0.31	0.11	0.41	0.05	T	4.35	0.07
						H	1.55	0.25
						TxH	0.60	0.46
Empty phagosome		0.34	0.07	0.53	0.01	T	0.80	0.40

					H	4.26	0.07
					TxH	0.46	0.52
Filled phagosome	0.06	0.01	0.10	0.06	T	0.64	0.47
					H	0.36	0.58
					TxH	1.88	0.24
Autophagosome	0.04	0.01	0.04	0.04	T	0.36	0.56
					H	0.00	>0.99
					TxH	1.46	0.26

Šídák's multiple comparisons test									
		1 h *vs* 24 h				Contra- *vs* Ipsi-			
Parameters	H	p	t	df	T	T	p	t	df
BBB contacts									
Basement membrane	C	0.36	1.39	8	1.00	1	0.13	2.52	8
	I	0.38	1.35		24.00	24	0.14	2.45	
Intracellular organelles									
Mitochondria	C	>0.99	0.00	8	1.00	1	0.08	2.94	4
	I	>0.99	0.00		24.00	24	0.08	2.94	
Dystrophic mitochondria	C	1.00	0.01		1.00	1	0.55	1.05	8
	I	0.44	1.24		24.00	24	0.10	2.30	
Primary lysosome	C	0.43	1.25		1.00	1	0.50	1.21	
	I	0.18	1.88		24.00	**24**	**0.04**	**3.83**	4
Secondary lysosome	C	0.49	1.15		1.00	1	0.37	1.51	
	I	0.29	1.56		24.00	24	0.11	2.65	

Table S9. Microglial ultrastructure relative frequency statistics.

p values are given after a Fisher's exact test measuring the relative frequency of ultrastructural features per cell across the ipsi- and contra- Lmol and the two timepoints (1 h and 24 h). For each data mean and S.E.M of n = 3 animals/timepoint are given. ANOVA: analysis of variance#: number, Lmol: stratum lacunosum moleculare, C or Contra- contralateral, I or Ipsi-: ipsilateral, p: p value and S.E.M. standard error of the mean.

			1 hour		24 hours			
			Contra-	Ipsi-	Contra-	Ipsi-	Fisher's exact test	
Parameter	unit		Mean	S.E.M	Mean	S.E.M	Group	p
Astrocytic endfeet containing mitochondria	#/cell	Mean					C1xI1	
		S.E.M					C24xI24	
Swollen astrocytic endfeet		Mean					C1xI1	
		S.E.M					C24xI24	
Extracellular space	Contact / cell	Mean	0.10	0	0.20	0.03	C1xI1	0.11
		S.E.M	0.05	0	0.06	0.03	**C24xI24**	**0.03**
Dystrophic mitochondria	#/cell	Mean	0.08	0.22	0.08	0.36	C1xI1	0.11
		S.E.M	0.04	0.07	0.04	0.08	**C24xI24**	**0.004**
Primary lysosome		Mean	0.18	0.19	0.26	0.05	C1xI1	>0.99
		S.E.M	0.06	0.07	0.07	0.04	**C24xI24**	**0.02**
Secondary lysosome		Mean	0.13	0.17	0.28	0.08	C1xI1	0.75
		S.E.M	0.05	0.06	0.07	0.05	**C24xI24**	**0.04**
Tertiary lysosome		Mean	0.34	0.25	0.18	0.25	C1xI1	0.45
		S.E.M	0.08	0.07	0.06	0.07	C24xI24	0.57

Table S10. Microglia ultrastructural nonlinear regression statistics.

p values are given after paired Wilcoxon test of Contra- *vs* Ipsi- nonlinear regression curve fits. These were modeled after the relative distribution of each parameter in the two *LMol* (contra- and ipsi-) nested into the two timepoints (1 h and 24 h). For each data, Amplitude, Mean and *p* value of n = 3 animals/timepoint/hemisphere are given. a.u.: arbitrary unit, *LMol*: *stratum lacunosum moleculare*, #: number, p: *p* value, Contra-: contra-, Ipsi-: ipsi- and h: hours.

		1 h		24 h	
		Contra-	Ipsi-	Contra-	Ipsi-
Synaptic plasticity contacts					
Synapse containing mitochondria contact (#/cell)	Amplitude	40.84	71.21	70.98	72.36
	Mean	-0.46	-3.54	-0.14	-0.21
	p	0.18		<0.0001	
Post-synaptic element (#/cell)	Amplitude	47.54	39.60	62.38	110.50
	Mean	0.96	0.20	0.61	0.44
	p	<0.0001		<0.0001	
Pre-synaptic element (#/cell)	Amplitude	29.22	39.60	28.87	27.76
	Mean	2.78	0.20	1.18	0.88
	p	<0.0001		<0.0001	
Intracellular organelles					
ER/Golgi (#/cell)	Amplitude	12.21	21.05	32.36	20.92
	Mean	7.43	4.29	3.97	4.41
	p	<0.0001		<0.0001	
Mitochondria #/cell (#/cell)	Amplitude	15.50	28.00	29.44	33.34
	Mean	0.11	-4.58	1.58	-0.12
	p	<0.0001		<0.0001	
Elongated mitochondria (#/cell)	Amplitude	89.95	56.09	65.89	106.40
	Mean	-0.89	-0.15	-0.25	-0.90
	p	<0.0001		<0.0001	

Table S11. Microglial ultrastructural principal component analysis.

For each PC, eigenvalue, proportion of variance, cumulative proportion of variance and loadings for each morphology parameter of n = 21 cells, N = 3 animals/timepoint/hemisphere are given. PC: principal components.

PC summary	PC1	PC2	PC3
Eigenvalue	3.252	3.026	2.363
Proportion of variance	12.51%	11.64%	9.09%
Cumulative proportion of variance	12.51%	24.14%	33.23%
Component selection	Selected	Selected	Selected
Loadings			
Astrocytic endfeet containing mitochondria contact	0.77	-0.07	0.19
Astrocytic endfeet contact	0.85	-0.15	0.14
Swollen astrocytic endfeet contact	0.62	-0.15	-0.14
Basement membrane contact	0.61	-0.07	0.10
Endothelial cell containing mitochondria contact	0.37	-0.13	0.15
Microglial blood vessel distance	-0.64	0.23	-0.10
Red blood cell contact	0.00	-0.07	-0.52
Synaptic element containing mitochondria contact	-0.38	0.09	0.61
Post-synaptic element contact	-0.28	-0.20	0.62
Pre-synaptic element contact	-0.15	-0.22	0.58
Myelinated axon contact	-0.23	-0.02	-0.20
Degenerating myelin contact	-0.03	0.00	-0.31
Satellite microglia	-0.03	-0.01	-0.39
Extracellular digestion	-0.13	-0.02	-0.35
Extracellular space contact	-0.02	-0.28	-0.17
ER/Golgi	-0.33	-0.77	0.03
Dilated ER	0.02	-0.49	-0.39
Mitochondria	-0.21	-0.69	0.06
Elongated mitochondria	-0.11	-0.63	0.27
Dystrophic mitochondria	-0.11	-0.45	-0.39
Primary lysosome	-0.15	0.04	-0.22
Secondary lysosome	-0.14	0.01	0.05
Tertiary lysosome	0.07	-0.66	0.06
Empty phagosome	0.20	-0.42	-0.06
Filled phagosome	-0.23	-0.44	-0.07
Autophagosome	-0.10	0.10	0.09

Table S12. Microglial ultrastructural correlation with blood vessel proximity and blood vessel area descriptive statistics.

p values are given after Spearman r correlation between blood vessel area and distance and ultrastructural parameters in the ipsi- *LMol*. For each dataset, r and p of n = 3 animals/timepoint/hemisphere are given. p: *p* value, r: r coefficient.

Ultrastructural parameters	Blood vessel distance		Blood Vessel area	
	r	p	r	p
Astrocytic endfeet containing mitochondria contact	-0.6213	0.0000	0.3151	0.0001
Astrocytic endfeet contact	-0.7330	0.0000	0.3624	0.0000
Swollen astrocytic endfeet contact	-0.3126	0.0019	0.3072	0.0001
Basement membrane contact	-0.4722	0.0000	0.1793	0.0276
Endothelial cell containing mitochondria contact	-0.2487	0.0146	0.1800	0.0270
Microglial blood vessel distance	1.0000	0.0000	-0.0669	0.5173
Vessel area	-0.0669	0.5173	1.0000	0.0000
Red blood cells contacts	-0.0662	0.5216	0.0921	0.2606
Post-synaptic contact	0.0774	0.4533	-0.0807	0.3246
Pre-synaptic contact	0.0082	0.9370	-0.0458	0.5768
Myelinated axon contact	0.0574	0.5787	-0.0635	0.4383
Degenerating myelin contact	0.0658	0.5244	-0.0221	0.7878
Satellite contact	0.0360	0.7274	-0.1194	0.1442
Extracellular digestion contact	0.1584	0.1231	0.0541	0.5097
Extracellular space contact	-0.0988	0.3380	0.0268	0.7436
ER/Golgi	0.0286	0.7819	0.0185	0.8215
Dilated ER	-0.0142	0.8906	0.0062	0.9398
Mitochondria	-0.0318	0.7585	0.0577	0.4814
Elongated mitochondria	-0.0336	0.7453	0.1197	0.1432
Dystrophic mitochondria	0.0254	0.8060	0.0312	0.7034
Mitochondria/ER	-0.0875	0.3966	0.0328	0.6894
Primary lysosome	0.0117	0.9096	-0.0906	0.2686
Secondary lysosome	0.0657	0.5248	-0.0029	0.9722
Secondary lysosome	-0.0512	0.6204	0.0262	0.7492
Empty phagosome	-0.0853	0.4088	0.0322	0.6948
Filled phagosome	0.0191	0.8533	0.0655	0.4241
Autophagosome	0.0134	0.8970	-0.0944	0.2487
